# Macitentan improves antitumor immune responses by inhibiting the secretion of tumor-derived extracellular vesicle PD-L1

**DOI:** 10.7150/thno.68864

**Published:** 2022-01-31

**Authors:** Chan-Hyeong Lee, Ju-Hyun Bae, Eun-Ji Choe, Ju-Mi Park, Seong-Sik Park, Hee Jin Cho, Byoung-Joon Song, Moon-Chang Baek

**Affiliations:** 1 Department of Molecular Medicine, CMRI, Exosome Convergence Research Center (ECRC), School of Medicine, Kyungpook National University, Daegu, Republic of Korea.; 2 Department of Biomedical Convergence Science and Technology, Kyungpook National University, Daegu, Republic of Korea.; 3 Section of Molecular Pharmacology and Toxicology, Laboratory of Membrane Biochemistry and Biophysics, National Institute on Alcohol Abuse and Alcoholism, NIH, Bethesda, MD, USA.

**Keywords:** Exosome, Extracellular vesicle, Cancer, immunotherapy, PD-L1

## Abstract

Extracellular vesicles (EVs) carrying tumor cell-derived programmed death-ligand 1 (PD-L1) interact with programmed death 1 (PD-1)-producing T cells, thus significantly lowering a patient's response to immune checkpoint blockade drugs. No drug that reinvigorates CD8+ T cells by suppressing EV PD-L1 has been approved for clinical usage. Here we have identified macitentan (MAC), an FDA-approved oral drug, as a robust booster of antitumor responses in CD8+ T cells by suppressing tumor cell-derived EV PD-L1.

**Methods:** EV was analyzed by the data from nanoparticle tracking, immunoblotting analyses, and nano-flow cytometry. Antitumor immunity was evaluated by luciferase assay and immune phenotyping using flow cytometry. Clinical relevance was analyzed using the cancer genome atlas database.

**Results:** MAC inhibited secretion of tumor-derived EV PD-L1 by targeting the endothelin receptor A (ETA) in breast cancer cells and xenograft models. MAC enhanced CD8+ T cell-mediated tumor killing by decreasing the binding of PD-1 to the EV PD-L1 and thus synergizing the effects of the anti-PD-L1 antibody. MAC also showed an anticancer effect in triple-negative breast cancer (TNBC)-bearing immunocompetent mice but not in nude mice. The combination therapy of MAC and anti-PD-L1 antibody significantly improved antitumor efficacy by increasing CD8+ T cell number and activity with decreasing Treg number in the tumors and draining lymph nodes in TNBC, colon, and lung syngeneic tumor models. The antitumor effect of MAC was reversed by injecting exogenous EV PD-L1. Notably, ETA level was strongly associated with the innate anti-PD-1 resistance gene signature and the low response to the PD-1/PD-L1 blockade.

**Conclusion:** These findings strongly demonstrate that MAC, already approved for clinical applications, can be used to improve and/or overcome the inadequate response to PD-1/PD-L1 blockade therapy.

## Introduction

Immune checkpoint blockade (ICB) therapy, which blocks checkpoints like programmed death 1 (PD-1)/programmed death-ligand 1 (PD-L1), has demonstrated a promising cancer immunotherapy with long-lasting efficacy. However, some patients respond to ICB therapy poorly due to various factors, such as tumor microenvironments (TME), tumor mutational burden, and systemic conditions 1-3. In particular, extracellular vesicles (EVs) have emerged as one of the key mechanisms regulating the overall response to the anti-PD-1/PD-L1 therapy 4-8. EVs include the exosomes secreted by various cells, including normal and tumor cells. EVs harbor proteins, lipids, RNAs, and DNA, and circulate in the body, thereby acting as important mediators in cancer initiation, progression, metastasis, and immunity 9-11. EV PD-L1 derived from tumor cells are primarily responsible for immune escape mechanisms, subsequently resulting in low responses to the PD-1/PD-L1 blockade therapy 4, 12. The EVs carrying PD-L1 inhibit the activity of T lymphocytes by binding to the PD-1 on their surface, leading to decreased efficacy of the anti-PD-L1 therapy 4, 13, 14. Thus, the EV-mediated inefficiency of ICB requires a new strategy to overcome its limitation possibly by controlling the biogenesis and secretion of PD-L1-containing EVs.

EV PD-L1 and decreased efficacy of anti-PD-1/PD-L1 therapy are causally related 4, 6, 12, 15-18. In colon and prostate cancer, the inhibition of EV PD-L1 from the tumor cells with a *Rab27a* or genetic deletion (knockdown, K/D) of neutral sphingomyelinase gene (*nSMase*) suppressed tumor growth in an immune-dependent manner 16. In addition, GW4869, which blocks EV secretion by inhibiting nSMase synthesis, synergizes with anti-PD-L1 therapy in 4T1 breast cancer-bearing mice 15. These results suggest that inhibiting EV PD-L1 can neutralize tumor immunosuppression and that this strategy may be used to enhance the efficacy of anti-PD-1/PD-L1 therapy.

Recently, we have demonstrated that endothelin receptor A (ETA) regulates the biogenesis and secretion of tumor-derived EVs 19. However, the EV components contributing to the antitumor effect in inhibiting tumor-derived EVs by ETA blockade are still unclear. Thus, we hypothesized that ETA-targeted drugs could reduce the number of EV PD-L1 and its binding with the PD-1 on T cells, thus inhibiting EV secretion, preventing tumor-mediated immune evasion, and ultimately improving the efficacy of anti-PD-L1 therapy. In addition, repositioning Food and Drug Administration (FDA)-approved ETA antagonists to inhibit the biogenesis of EV PD-L1 can be quickly applied in clinics without serious side effects.

In this study, we focused on investigating the immunological function of tumor-derived EV PD-L1 suppressed by ETA blockade *in vitro* and *in vivo*. We have observed that macitentan (MAC), an FDA-approved ETA antagonist, effectively inhibits the secretion of EV PD-L1 and enhances the tumor-killing effect of CD8^+^ T cells 20. Our results confirmed that the coadministration of MAC and an anti-PD-L1 antibody improves antitumor immunity and memory in triple-negative breast cancer (TNBC), colon cancer, and lung cancer models.

## Materials and Methods

### Chemicals

Macitentan (MedChemExpress), recombinant human interferon-γ (IFN-γ, PeproTech), sulfisoxazole (SFX) and BQ123 (Sigma-Aldrich), and bosentan, ambrisentan, and BQ788 (Tocris Bioscience) were purchased.

### *In vivo* blocking antibodies

InVivoMab anti-mouse PD-L1 (B7-H1) and InVivoMab rat IgG2a isotype control were diluted in InVivoPure pH 7.0 Dilution Buffer (Bio X Cell, Lebanon, NH, USA).

### Cell lines and cell culture

All human cancer and murine cancer cells (American Type Culture Collection) were grown at 37 °C under a humidified atmosphere with 5% CO_2_ and 95% air. MDA-MB231 and 4T1 cells were cultured in Dulbecco's modified Eagle's medium with 10% fetal bovine serum (FBS) and 1% antibiotic-antimycotic solution. CT26 and LL/2 cells were cultured in RPMI with 10% FBS and 1% antibiotic-antimycotic solution. EMT6 cells were cultured in Waymouth's MB 752/1 medium with 2 mM L-glutamine, 15% FBS, and 1% antibiotic-antimycotic solution. For the analysis of EV inhibition, the cells were washed and incubated in an FBS-free medium. Human CD8^+^ T cells from the peripheral blood mononuclear cells (PBMCs) from healthy donors were cultured in RPMI with 20% FBS 1% antibiotic-antimycotic solution, and 100 IU/ml human IL-2. All the cell lines were tested for mycoplasma contamination using PCR genotyping.

MDA-MB231, 4T1, and CT26 cell lines were transfected with human Control shRNA Lentiviral Particles, human ETA Lentiviral Particles, mouse Control shRNA Lentiviral Particles, and mouse ETA Lentiviral Particles (OriGene Technologies, Inc., Rockville, MD, USA).

### Isolation and quantitation of EV

EVs were purified using differential centrifugation 19, 21. The supernatants from cell cultures were sequentially centrifuged at 300 × *g* for 3 min, 2,500 × *g* for 15 min, and 10,000 × *g* for 30 min. After passing through a 0.22-μm filter, the supernatants were centrifuged at 120,000 × *g* for 90 min. The EV pellets were resuspended with PBS, centrifuged at 120,000 × g for 90 min, and homogenized in PBS or 1X RIPA buffer for additional analyses.

Mouse plasma was centrifuged at 2,500 × *g* for 15 min and 10,000 × *g* for 30 min to remove the intact cells and cell debris. The supernatant was then centrifuged at 120,000 × g for 90 min. EV proteins were quantified using the Pierce BCA Protein Assay kit (Thermo Scientific) after homogenization in RIPA buffer (Cell Signaling Technology, CST).

### Transmission electron microscopy analysis

Purified EVs were deposited onto pure carbon-coated EM grids. For immunogold labeling, EV were incubated with anti-human PD-L1 antibody (eBioscience), then with an anti-mouse IgG conjugated with 5-nm gold particles (Sigma-Aldrich). After staining with 2% uranyl acetate, the grids were dried at 25 ºC and visualized at 6,000 × and 40,000 × using the HT-7700 transmission electron microscope (Hitachi) operated at 100 kV.

### Cell viability assay

Drug-induced cell cytotoxicity was measured using the MTT [3-(4,5-dimethylthiazol-2-yl)-2,5-diphenyltetrazolium bromide] assay. First, the cells were seeded in a 24-well plate at a density of 2 × 10^4^ cells/well and grown for 24 h. Then, the culture medium was replaced with an FBS-free medium with different concentrations of drugs, and the cells were grown for another 24 h. Next, a fresh medium with 500 μg/ml MTT was added to each well, and the cells were incubated at 37 °C for 3 h. After removing the supernatant, 200 μl of dimethyl sulfoxide was added to each well to dissolve the violet formazan crystals, and the optical density of each well was assessed with a microplate reader at 550 and 570 nm.

### Western blot assay

Cellular or EV proteins were resolved by SDS-PAGE, transferred onto nitrocellulose membranes, and probed with the respective primary antibody against CD63 (1:1000; Abcam), beta-actin (1:20000; CST), ALIX (1:1000; CST), flotillin-1 (1:1000, CST), RAB27A (1:1000; Abcam), ETA (1:2000; Abcam), Akt (1:2000; CST), phospho-Akt (1:2000; CST), or PD-L1 (1:1000; CST). After removing the primary antibody and three washing steps at 10-min intervals, the blots were incubated with a horseradish peroxidase (HRP)-linked secondary antibody. The images were visualized using enhanced chemiluminescence (ECL) detection reagents (Thermo Scientific) and quantified using ECL hyper-film (AGFA) and Fusion FX7 system (Vilber Lourmat).

### Immunohistochemistry on tumor sections

The tumor tissues were fixed in 4% neutral formaldehyde for 48 h, embedded in paraffin, sliced into 4-μm sections, and stained with an anti-mouse CD8α antibody (CST). The slides were then incubated using EnVision+/HRP rabbit antibody (Dako) for 30 min at room temperature to obtain immunohistochemical images.

### Nano-flow cytometry

EV PD-L1 was analyzed using nano-flow cytometry 22. The EVs from mouse plasma were incubated with allophycocyanin (APC)-labeled anti-human PD-L1 antibody (eBioscience) for 2 h at room temperature in the dark and ultracentrifuged at 120,000 × g for 90 min to remove the unbound antibodies. Nano-flow cytometry was performed using the CytoFLEX system (Beckman Coulter). The 405-nm violet laser for side scatter (V-SSC) was selected with the manual threshold setting of 2000 in the V-SSC height channel. For calibration, 100, 200, and 400-nm polystyrene beads were used. The EVs were gated on the size of 100-200 nm. Data were acquired and analyzed using CytExpert 2.3 (Beckman Coulter).

### Nanoparticle tracking analysis

The EVs were counted using the nanoparticle tracking analysis (NTA) 19. EV suspensions from cell culture medium were analyzed using the LM10 instrument (NanoSight). A 405-nm monochromatic laser beam was applied to a diluted suspension of EV. Thirty-second videos were recorded with the rate of 30 frames/s, and EV movement was analyzed using the NTA software version 2.2 (NanoSight). Post-acquisition settings of the NTA were optimized and maintained between samples, and each video was analyzed to estimate the EV concentration.

### PD-1 binding assay

The binding of EV PD-L1 to PD-1 was examined as described previously 4. In summary, 96-well ELISA plates were coated with 4 µg/ml human PD-1 protein (BPS Bioscience) overnight at 4 °C. After removing excess and unbound PD-1 protein, free binding sites were blocked with phosphate-buffered saline (PBS) containing 0.05% Tween 20 (PBS-T) for 2 h at room temperature. Then 100 µl EVs per well were incubated overnight at 4 °C. After washing, 100 µl of 4 µg/ml biotin-labeled anti-PD-L1 antibody (eBioscience) was added, and the mixture was incubated for 2 h at room temperature. Next, 100 µl of horseradish peroxidase-conjugated streptavidin (BD Biosciences) was added per well, and the mixture was incubated for 1 h at room temperature. The chromogenic signals were developed using 3,3′,5,5′-tetramethylbenzidine-containing peroxide for 30 min. The reaction was stopped, and optical density in each well was measured at 450 nm using an automated iMark (Bio-Rad).

### Isolation of CD8^+^ T cells and treatments with the EVs

CD8^+^ T cell activities were measured by the method reported earlier 4. Purified EVs were incubated with 10 μg/ml anti-PD-L1 antibodies in PBS, washed with 30 ml PBS, and pelleted by ultracentrifugation to remove the free antibodies. Human CD8^+^ T cells were purified from the PBMCs using a human CD8^+^ T cell isolation kit (Miltenyi Biotec). The CD8^+^ T cells were activated by incubation with 2 µg/ml human CD3/CD28 antibody and 100 IU/ml human IL-2 for 24 h and then with MDA-MB231-derived EVs with or without PD-L1 blocking for 48 h in the presence of the anti-CD3/CD28 antibodies and IL-2.

### CD8^+^ T cell-mediated cytotoxicity assay

Human CD8^+^ T cells were purified from the PBMCs of healthy donors. First, the CD8^+^ T cells were activated by incubation with 2 µg/ml human CD3/CD28 antibody for 24 h. Next, MDA-MB231-luciferase cells were seeded into 96-well plates at 5,000 cells/well. After 12 h, the MDA-MB231-luciferase cells were treated with or without drugs or EV-containing media and then cocultured with human CD8^+^ T cells for 48 h at an effector to target (E:T) ratio of 1:1. The plates were washed with PBS, and 100 µl of 2 mg/ml luciferin was added to each well. The luciferase intensity in each well was immediately measured using an Alpha Microplate reader (PerkinElmer).

### Immune phenotyping using flow cytometry

The tumors were harvested, mechanically cut into small pieces of less than 4 mm in length, and enzymatically digested using a mouse tumor dissociation kit (Miltenyi Biotec) for 45 min at 37 °C. CD45^+^ T cells were isolated with mouse CD45 MicroBeads (Miltenyi Biotec). Red blood cells from the spleen were lysed using the Red Blood Cell Lysing Buffer (Sigma). The dead cells isolated from the tumor, DLN, and spleen were stained for 30 min on ice with the Fixable Aqua Dead Cell Stain Kit (Invitrogen). The samples were first stained for surface markers of the immune cell populations. For intracellular staining, the Foxp3/Transcription Factor Staining Buffer Set (eBioscience) was used. The samples were blocked for 10 min on ice with Fc block (BD Pharmingen) before antibody staining. The fluorescein isothiocyanate (FITC) anti-mouse CD3, phycoerythrin (PE) anti-mouse ki67, PE anti-mouse PD-1 antibodies (BioLegend), the efluor780 anti-mouse CD45, peridinin chlorophyll protein-Cyanine 5.5 anti-mouse CD4, efluor450 anti-mouse CD8, APC anti-mouse GzmB, PE anti-mouse FoxP3 and APC anti-mouse Tim3 (eBioscience) were used. The immune phenotyping was performed using the CytoFLEX system (Beckman Coulter). Single-dye stains were performed for compensation controls, and the level of nonspecific binding was evaluated with isotype controls.

### Animal study

Six to seven-week-old *nude* (BALB/cAnNCrl-nuBR), BALB/c (BALB/cAnNCrl) and C57BL6/J (C57B6NCrl) mice were purchased from Orient Bio (Seongnam, Korea). Mice were bred and maintained in a specific pathogenfree barrier facility. For the analysis of circulating EV PD-L1, 2 × 10^6^ MDA-MB231 cells suspended in 100 µl of PBS with 50% Matrigel (Corning) were orthotopically injected into the left fat pad of the female BALB/c *nude* mice. After 21 days, the mice were euthanized, and the blood plasma and tumors were isolated. In the syngeneic tumor models, 4T1 and EMT6 breast tumor cells, at 2 × 10^5^ and 2 × 10^5^ cells, respectively, in 100 µl of PBS with 50% Matrigel, were orthotopically injected into the left fat pad of the female *nude* or BALB/c mice. The CT26 colon tumor cells, at 2 × 10^5^ cells in 100 µl PBS, were subcutaneously injected into the right flank of male* nude* or BALB/c mice. The LL/2 lung tumor cells, at 2 × 10^5^ cells in 100 µl of PBS, were subcutaneously injected into the right flank of male* nude* or C57BL6/J mice. During the animal studies, tumor volumes were recorded until they reached the maximum value defined by IACUC guidelines. When the tumors reached an average size of 50-100 mm^3^, the mice were randomized into groups with a comparable distribution of starting tumor volumes. The mice were euthanized when their tumor volumes reached 1500 mm^3^.

Tumor growth was measured every 3-4 days using a caliper. The tumor size was estimated using the equation: volume (cm^3^) = width^2^ × length × 0.5. MAC was orally administered at 50 mg kg^-1^ day^-1^ for 14-25 days. Anti-mouse PD-L1 antibody and rat IgG2a isotype control were intraperitoneally injected once every 3 days for 3 times. Complete regression was defined as the tumors below 50 mm^3^ and continuing to regress until the end of the study. For analysis of metastasis, 4T1-luciferase-inoculated mice were monitored using an IVIS imaging system (Perkin Elmer). In the rescue experiments, 5 μg of EMT6 and CT26-derived EVs were intravenously injected into tail vein once every 3 days for 3 times.

### Analysis of gene expression in human cancer databases

Tumor sample gene expression data were obtained from TCGA, including 1082 breast cancer patients. CIBERSORT (https://cibersort.stanford.edu) was used to estimate tumor immune cell infiltration. The correlation between *EDNRA* expression and the innate anti-PD-1 resistance (IPRES) gene signature was analyzed 23. Gene signatures previously associated with IPRES were obtained from Broad MSigDB (http://software.broadinstitute.org/gsea/msigdb) and the original publication. The gene set variation analysis scores of the signatures were computed for the TCGA patient samples, transformed to z-scores, and correlated with *EDNRA* expression by single sample gene set enrichment analysis (ssGSEA) in gene set variation analysis (GSVA) version R package. The gene expression data from patients responding to anti-PD-1, including 28 melanoma samples, were obtained from the gene expression omnibus (GEO) database.

### Statistical analysis

Statistical analyses were performed using GraphPad Prism version 6.0. Unpaired two-tailed Student's *t*-test was used to compare two sets of data. The error bars in the graphical data represent means ± standard deviation. Tumor volume and immune phenotyping data were presented as means ± SEM. All the *in vitro* experiments were performed in triplicates unless otherwise stated. The *p*-values less than 0.05 were considered to denote statistically significant differences; *, **, ***, and **** represent a* p*-value of less than 0.05, 0.01, 0.001, and 0.0001, respectively.

## Results

### MAC suppresses EV PD-L1 secretion *in vitro* and* in vivo*

**Figure 1:**
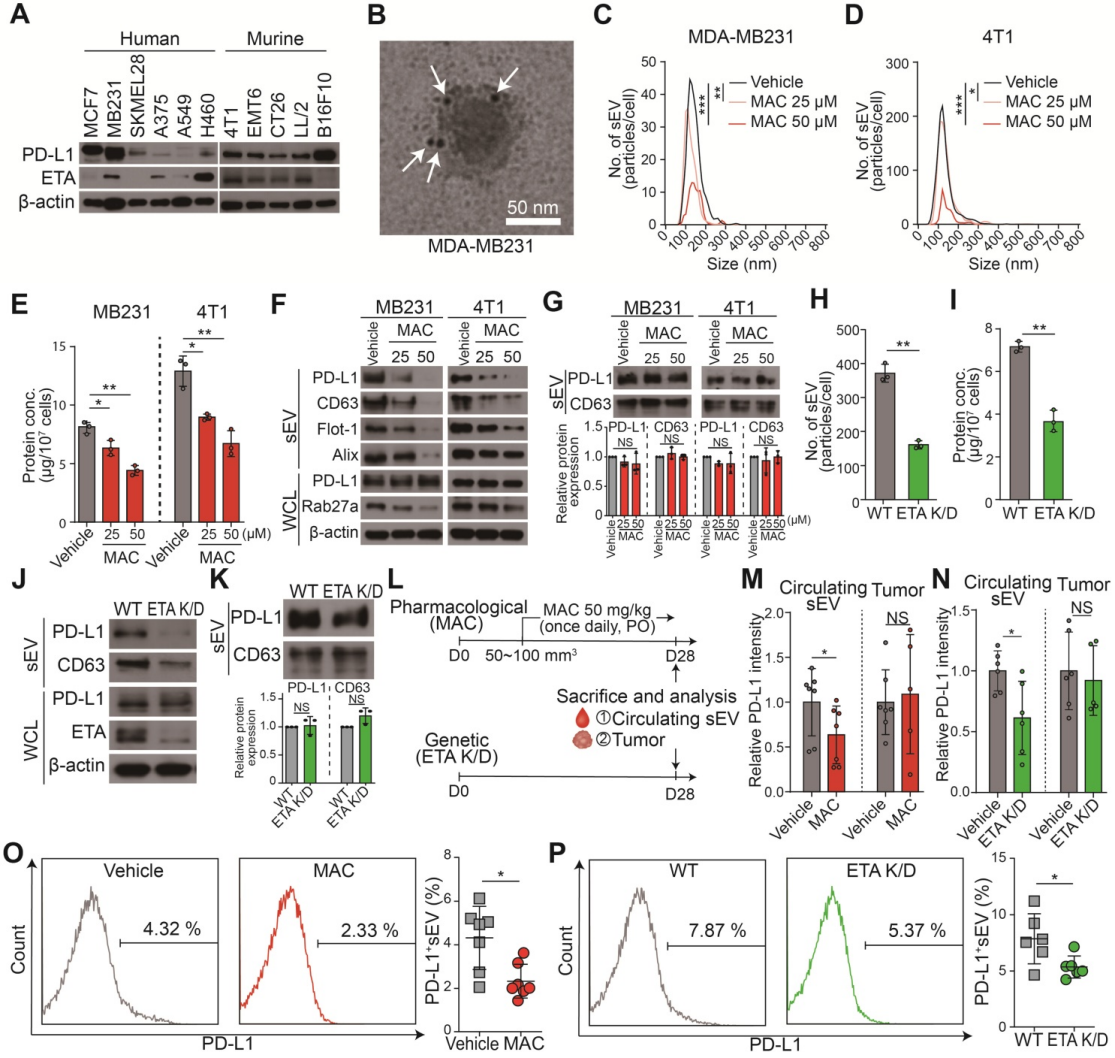
** MAC suppresses EV PD-L1 by targeting ETA *in vitro* and *in vivo*. (A)** The immunoblots of PD-L1 and ETA in the indicated cancer cell lines. **(B)** A transmission electron microscopy (TEM) image of MDA-MB231-derived EVs immunogold-labeled with anti-PD-L1 antibodies. Arrowheads indicate 5 nm gold particles. Scale bar, 50 nm. **(C)** The number of EVs derived from MDA-MB231 cells and **(D)** 4T1 cells with or without MAC treatment by nanoparticle tracking analysis (NTA). **(E)** The amounts of protein in the EVs derived from MDA-MB231 and 4T1 cells in the absence or presence of MAC. **(F)** The immunoblot of various proteins in EV and whole-cell lysates from MDA-MB231 and 4T1 cells with or without MAC (*n* = 3). EV proteins from 1 × 10^7^ cells were loaded per lane. **(G)** The immunoblot of PD-L1 and CD63 in the EVs from MDA-MB231 and 4T1 cells treated with or without MAC at the indicated concentration (top). The densitometric analysis of the relative intensity of the protein bands (bottom) (*n* = 3). **(H)** The number of and **(I)** amount of protein in the EVs derived from the wild-type (WT) and ETA K/D MDA-MB231 cells. **(J)** The immunoblot of various proteins in EV and whole-cell lysates from the WT or ETA K/D MDA-MB231 (*n* = 3). Beta-actin was used as the loading control. EV proteins from 1 × 10^7^ cells were loaded per lane. **(K)** The immunoblot of PD-L1 and CD63 in the EVs from WT or ETA K/D MDA-MB231 cells (top) (*n* = 3). The densitometric analysis of the relative intensity of the protein bands (bottom). An equal amount of protein (5 µg) of the EVs was loaded per lane. **(L)** The experimental designs using the MDA-MB231 xenograft models. **(M)** Densitometric analysis of the immunoblot of PD-L1 in circulating EVs and tumor lysates from MDA-MB231 xenograft mice treated with or without MAC (left,* n* = 5 and 7, respectively) and **(N)** in WT or ETA K/D MDA-MB231 xenograft mice (left,* n* = 5 and 6, respectively), related to figure S2A and B. **(O)** The level (%) of surface PD-L1 of the EV derived from the plasma of the MDA-MB231 xenograft model with or without MAC (*n* = 7) and **(P)** in WT or ETA K/D MDA-MB231 xenograft mice* (n* = 6). The data are presented as means ± SD. *p < 0.05, ** p < 0.01, ***p < 0.001, and **** p < 0.0001, respectively; NS, not significant.

We hypothesized that ETA antagonists reduced the binding of EV PD-L1 to the PD-1 on CD8^+^ T cells by inhibiting the biogenesis and secretion of EV. Thus, the FDA-approved ETA antagonist drugs (e.g., MAC, bosentan, SFX, and ambrisentan) were screened for their inhibitory effect on EVs. First, the high protein levels of PD-L1 and ETA in MDA-MB231 human and 4T1 murine TNBC cells and PD-L1 protein in the MDA-MB231 cell-derived EVs were confirmed (Figure 1A-B). Noncytotoxic concentrations of the ETA antagonists were used to test and confirm their inhibition of EV secretion (Figure S1A). All the antagonist drugs tested inhibited endothelin-2 (ET2) mediated ETA signaling and EV PD-L1 secretion (Figure S1B-D). MAC most potently inhibit the levels of EV PD-L1 than other ETA antagonists. In addition, we examined whether the blocking of endothelin receptor B (ETB) affected the secretion of EVs. Our results showed that ETA antagonist BQ123, but not ETB antagonist BQ788, inhibited EV secretion, suggesting an ETA-specific secretion of EVs (Figure S1E).

Next, we investigated the effect of MAC on the levels of EV-related proteins in secreted EVs and whole cell lysates (WCL) from MDA-MB231 and 4T1 cells. Consistent with reduced EV secretion, the amounts of PD-L1 and EV marker proteins, such as CD63, filotillin-1 (Flot-1), and ALIX, decreased in EVs from both cells following MAC exposure (Figure 1C-F). In the WCL of MAC-treated cells, RAB27A, a main regulator of EV secretion, decreased as expected. However, there was no significant change in PD-L1 protein level in the WCL of both cells (Figure 1F). Based on the results that MAC did not change the protein levels of PD-L1 and CD63 in the same protein amount of EVs (Figure 1G), we confirmed that MAC inhibited EV PD-L1 by inhibiting EV secretion without impediment of PD-L1 sorting from tumor cells to EV. Similar to the effects of MAC, downregulated endothelin receptor A (*EDNRA*) expression in the ETA K/D cells reduced EV PD-L1 but did not alter the level of cellular PD-L1 (Figure 1H-J) or the amounts of PD-L1 and CD63 in the same protein amount EVs (Figure 1K). These results suggested that MAC inhibits the secretion of EV PD-L1 by antagonizing ETA and that the reduction of EV PD-L1 by MAC is due to the inhibition of total EV secretion from tumor cells.

The MDA-MB231 xenograft model was used to determine whether the amounts of circulating EV PD-L1 from tumor cells were decreased by MAC (Figure 1L). First, it was confirmed that the PD-L1 level of plasma EV was increased in tumor-bearing mice (Figure S2A). Next, we determined the administration route and dosage of MAC. MAC was developed as a tablet for oral use in patients with pulmonary arterial hypertension (PAH) and was administered orally in many studies 24-28. Additionally, a 50 mg/kg dose of MAC was selected for the animal study because this dose was used for cancer therapy 24-28, and no serious toxicity was reported at a dose of 50 mg/kg daily for 4 weeks 25. EV PD-L1 in plasma was significantly suppressed in the MAC group compared to the vehicle group (Figure 1M, S2B). In contrast, there was no change in PD-L1 protein level in the tumor lysates from the MAC-treated mice. These results were consistent with the *in vitro* findings that MAC reduced the secretion of tumor-derived EV PD-L1 without affecting PD-L1 expression in tumor cells. In ETR K/D MDA-MB231 xenograft, the level of EV PD-L1 were also decreased, although the protein level of PD-L1 was unchanged in the tumor lysate (Figure 1N, S2C). In addition, nano-flow cytometry showed that the decrease amounts of EV PD-L1 from the plasma were consistently observed in the ETA K/D mice and MAC-treated xenograft model (Figure 1O-P, S2D). These results demonstrate that MAC inhibits the tumor-derived EV PD-L1 by antagonizing ETA of tumor cells *in vivo*.

### MAC boosts T cell cytotoxicity activity by inhibiting EV PD-L1

**Figure 2:**
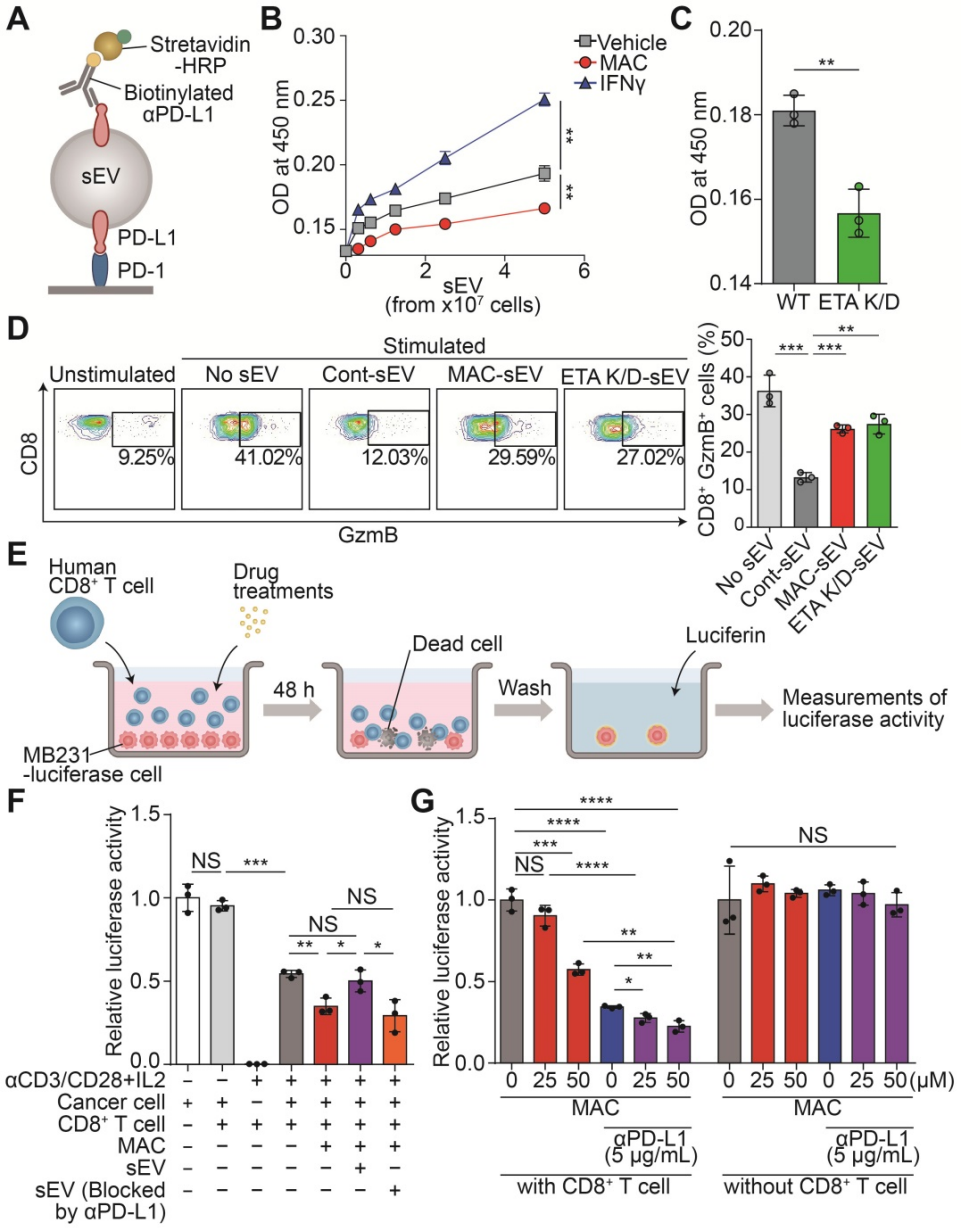
** MAC boosts T cell-mediated cytotoxic activity by suppressing EV PD-L1. (A)** The scheme of the PD-1 binding assay. **(B)** The measurement of PD-1 binding with EV PD-L1 derived from MDA-MB231 cells treated with (50 µM) MAC or (50 ng/ml) IFN-γ. **(C)** The measurement of PD-1 binding with EV PD-L1 derived from WT or ETA K/D MDA-MB231 cells. **(D)** The human CD8^+^ T cells with the indicated treatments were examined for the GzmB level by flow cytometry (left), showing the proportions of the GzmB-positive cells (right). **(E)** The experimental designs of the coculture system to test the CD8^+^ T cell activity. **(F)** The human CD8^+^ T cell-mediated cytotoxicity in the MDA-MB231-luciferase cells after the indicated treatments. **(G)** Luciferase activity of the MDA-MB231-luciferase cells with or without human CD8^+^ T cells after the indicated treatments. The data are presented as means ± SD (*n* = 3). *p < 0.05, **p < 0.01, ***p < 0.001, and ****p < 0.0001, respectively; NS, not significant.

EV-mediated immunosuppression is believed to result from the interaction between PD-L1 on the EV and the PD-1 on the surface of CD8^+^ T cells. Thus, we performed a PD-1 binding assay to determine whether MAC treatment resulted in less binding between PD-1 and the PD-L1 in the EVs secreted from MDA-MB231 cells (Figure 2A). Interferon-γ (IFN-γ), used as a positive control, increased only PD-L1 levels without changing the amounts of secreted EVs (Figure S3A-B), and the binding to PD-1 increased in the EV concentration-dependent manner (Figure 2B). In contrast, the EVs derived from MAC-treated or ETA K/D cells had significantly decreased PD-1 binding compared to the vehicle group (Figure 2B-C). This appears to be a phenomenon that occurs because the number of EVs secreted from cells decreases without a corresponding change in PD-L1 protein levels on EVs (Figure 1B-E, S3A-B). The reduction in the number of EV PD-L1 from cancer cells owing to MAC treatment might increase the activity of T cells *in vivo*.

Next, the CD8^+^ T cells isolated from human peripheral blood mononuclear cells (PBMCs) were analyzed to determine whether the MAC-mediated inhibition of EV PD-L1 affects the activity of the CD8^+^ T cells (Figure S3C). After the T cells were treated with MDA-MB231-derived EVs, the level of granzyme B (GzmB), a cytotoxic marker, decreased in a concentration-dependent manner. However, the EVs, blocked with anti-PD-L1 antibody treatment, did not have the same effect (Figure S3D). Next, we tested whether the reduction of EV PD-L1 by MAC treatment could increase CD8^+^ T cell activity. The EVs derived from MAC-treated cells or ETA K/D cells increased T cell activity compared to that with the EVs derived from the vehicle-treated cells (Figure 2D).

Further, we verified that the MAC-induced activation of CD8^+^ T cells increased their tumor-killing function in a coculture system (Figure 2E). First, we confirmed the luciferase activity of the MDA-MB231-luciferase cells positively correlated with their cell numbers (Figure S3E). We observed cytotoxic activity by the activated CD8^+^ T cells in the coculture of CD8^+^ T cells and MDA-MB231 cancer cells. In fact, MAC treatment increased the CD8^+^ T cell-mediated cytotoxic effect (Figure 2F). We also observed that treatment of EV PD-L1 derived from MDA-MB231 cells restored cancer cell death induced by MAC (Figure S3F). However, treatment of EVs, blocked by the presence of anti-PD-L1 antibody, still showed a cytotoxic effect by MAC (Figure 2F). These results suggest that the increase in CD8^+^ T cell mediated cytotoxic activity is due to the inhibition of EV PD-L1 by MAC treatment. In addition, the combined treatment of MAC with anti-PD-L1 antibody augmented cancer cell death without direct cytotoxic effect of these two drugs on the MDA-MB231 cells (Figure 2G). Moreover, MAC did not affect the viability or activity of the CD8^+^ T cells (Figure S3G-H). These results suggest that the inhibition of EV PD-L1 by MAC augments the CD8^+^ T cell-mediated cytotoxic effect and significantly increases the efficacy of the PD-1/PD-L1 blockade.

### MAC enhances the efficacy of anti-PD-L1 therapy

We investigated whether MAC could enhance the anti-tumor effects by immune checkpoint blockade (ICB) in immunocompetent animals. We studied several mouse cancer models, including TNBC (4T1, EMT6), colorectal (CT26), and lung (LL/2). Considerable amounts of ETA and PD-L1 proteins were detected in the mouse cells (Figure S1A) and the significant suppression of EV PD-L1 by MAC was observed (Figure S4A-B).

**Figure 3:**
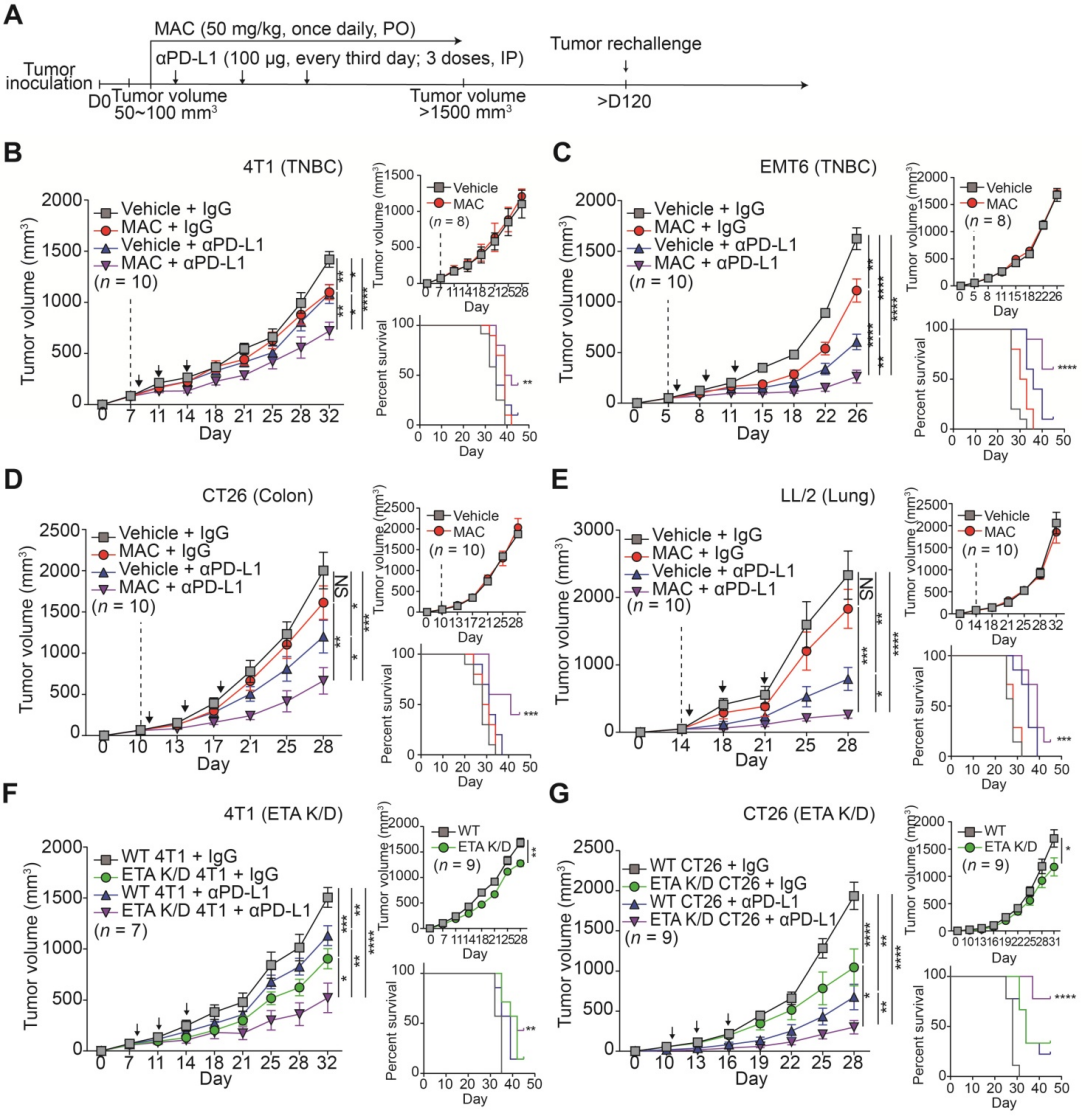
** MAC augments the efficacy of the anti-PD-L1 antibody therapy in multiple syngeneic cancer models. (A)** The experimental designs of the syngeneic cancer models. **(B-E)** The growth curves of (B) 4T1 and (C) EMT6 breast cancer, (D) CT26 colon cancer, and (E) LL/2 lung cancer tumors in immunocompetent mice (left, *n* = 7-10) and nude mice (top right, *n* = 8-10) and the survival rate (bottom right) of the mice treated with the vehicle or MAC and isotype IgG or anti-PD-L1 antibody. The vertical dotted lines indicate the beginning of MAC administration (at tumor volume = 50-100 mm^3^). The blue arrows represent the timing of anti-PD-L1 antibody administrations. **(F)** The growth curves of WT and 4T1 ETA K/D or **(G)** CT26 ETA K/D tumors in immunocompetent mice (left,* n* = 7-9) and nude mice (top right,* n* = 9) and the survival rate of each group (bottom right). The dotted line indicates the beginning of the anti-PD-L1 antibody treatment (at tumor volume = 50-100 mm^3^). The survival rates were analyzed using the Mantel-Cox test. The data are presented as means ± SEM. *p < 0.05, **p < 0.01, ***p < 0.001, and ****p < 0.0001, respectively; NS, not significant; Mann-Whitney U test.

We determined the effect of MAC on the immune system by monitoring the tumor volumes in the immunocompetent mice and the nude mice with impaired T lymphocyte development (Figure 3A-E, S5A-D). MAC alone showed an antitumor effect only in immunocompetent mice but not in nude mice from the 4T1 and EMT6 breast cancer models (Figure 3B-C, S5A-B). In the CT26 and LL/2 mice, the antitumor effect of MAC was not observed in the immunocompromised or immunocompetent mice (Figure 3D-E, S5C-D). Nevertheless, in all four mouse models, the coadministration of MAC with anti-PD-L1 antibody showed a stronger antitumor effect and higher survival rate than the vehicle, MAC, or anti-PD-L1 antibody alone (Figure 3A-E, S5A-D). In addition, the reduction of EV secretion by MAC inhibited metastasis in the 4T1 syngeneic tumor model (Figure S6A-B).

Next, we studied the efficacy of the anti-PD-L1 antibody in the ETA K/D models. The 4T1 or CT26 ETA K/D cells were constructed using shRNA-inhibited EV PD-L1 (Figure S4C-E). In nude mice, the 4T1 and CT26 ETA K/D models showed 24% and 31% total growth inhibition (TGI), respectively. The same models exhibited stronger growth inhibition, by 65 and 40%, respectively, in immunocompetent mice (Figure 3F-G, S5E-F). Additionally, anti-PD-L1 antibody administration to the ETA K/D mice showed a stronger antitumor effect and higher survival rate than the corresponding WT mice. These results indicate that targeting ETA with MAC inhibits tumor growth in immunocompetent patients and enhances the efficacies of anti-PD-L1 antibody therapy.

To determine whether MAC with an anti-PD-L1 antibody induces immunological memory, the mice with tumor regression, following the combination therapy in the EMT6 and CT26 syngeneic tumor models, were rechallenged with another set of tumor (Figure 3A). Age-matched, tumor-naïve WT mice were used as controls (Figure S7A-B). In the control group, tumors grew in a time-dependent manner, whereas the mice with regressed tumors following the combination therapy remained tumor-free. Thus, combined treatment with MAC and an anti-PD-L1 antibody likely induces long-term immune memory in multiple tumor models.

### Combination of MAC and anti-PD-L1 antibody boosts antitumor immune responses

We used EMT6 tumor-bearing mice, which demonstrated the strongest response to the combination therapy (Figure 3C), to further study the underlying mechanisms by which the combined therapy affected the immune system. For this purpose, the effect of MAC, anti-PD-L1, or both on tumor growth was studied (Figure 3C); tumor volume was significantly reduced at 22 days (Figure S8A-B). In addition, the amounts of PD-L1, but not CD63, in the EVs from the plasma of the mice treated with MAC alone or with combination of MAC and anti-PD-L1 antibody were significantly decreased, compared to the vehicle or anti-PD-L1 alone group (Figure S8C), suggesting that MAC inhibited tumor-derived EV PD-L1 *in vivo*.

**Figure 4:**
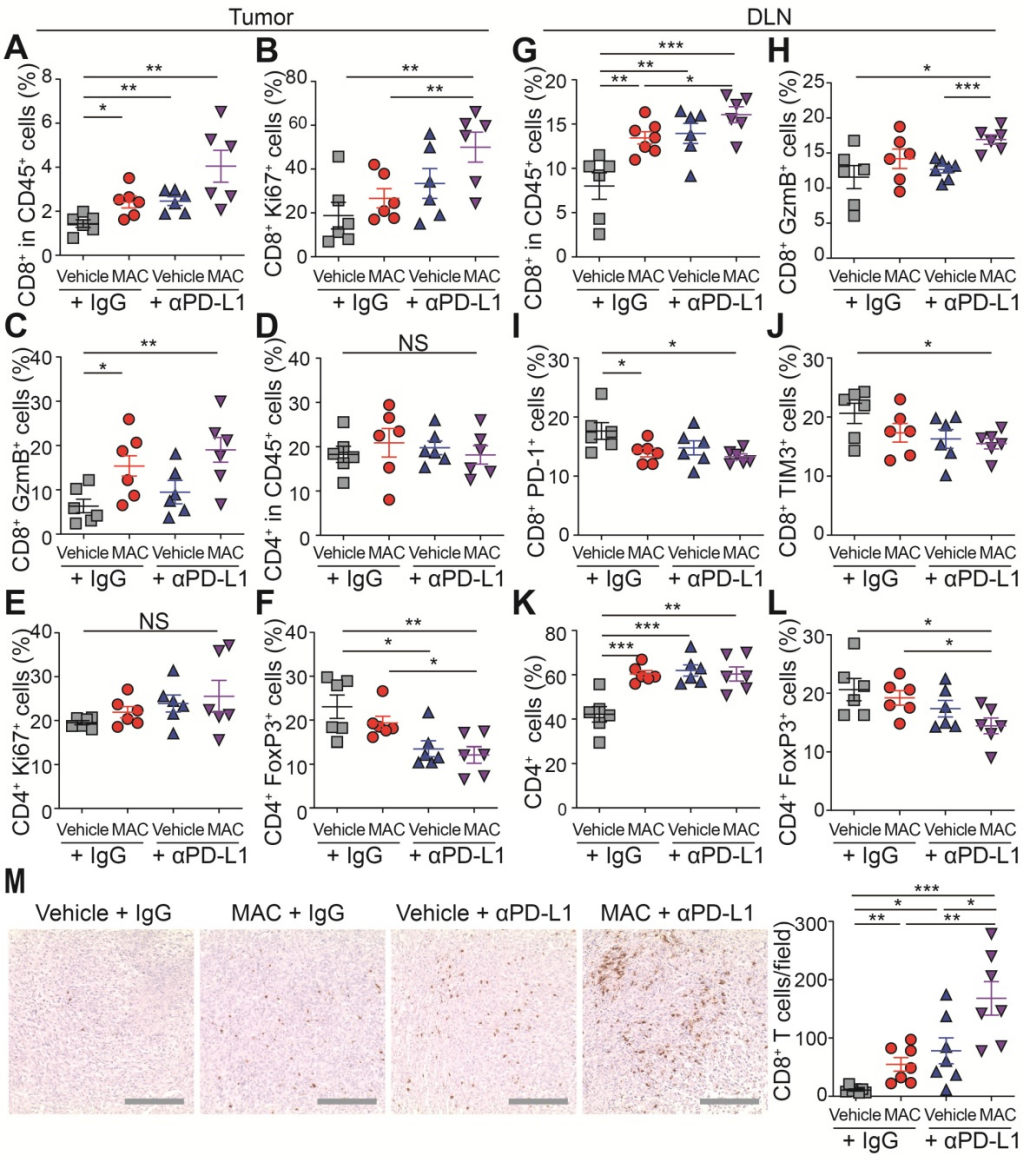
** The combination therapy of MAC and anti-PD-L1 antibody increases immune response in EMT6 tumor model. (A-M)** Flow cytometry analysis of the T lymphocyte in tumors and DLN (*n* = 6). The proportions of CD8^+^ cells in the (A) CD45^+^ cells, (B) ki67^+^ cells, and (C) GzmB^+^ cells in the tumor. The proportions of CD4^+^ cells in the (D) CD45^+^ cells, (E) ki67^+^ cells, and (F) FoxP3^+^ cells in the tumor. The proportions of CD8^+^ cells in the (G) CD45^+^ cells, (H) GzmB^+^ cells, (I) PD-1^+^ cells, and (J) Tim3^+^ cells in the DLN. The proportions of CD4^+^ cells in the (K) CD45^+^ cells and (L) FoxP3^+^ cells in the DLN. (M) IHC Images of anti-CD8^+^ T cells in tumors (left, *n* = 7) and the percentages of CD8^+^ T cells (right). Scale bar, 100 µm. The data are presented as means ± SEM. *p < 0.05, **p < 0.01, and ***p < 0.001, respectively; NS, not significant; Mann-Whitney U test.

Next, we studied the immunophenotype of the tumor and draining lymph node (DLN) (Figure 4A-L, S9A-B). The population, proliferation and cytotoxic activity of CD8^+^ T cells were significantly increased in the tumor of the mice co-treated with MAC and anti-PD-L1 antibody (Figure 4A-C), whereas the population and proliferation of CD4^+^ T cells were unchanged (Figure 4D-E). In addition, the proportion of regulatory T cells (Tregs), potent inhibitors of CD8^+^ T cells, was significantly decreased in the combination therapy group compared to the vehicle group (Figure 4F). In DLNs, as in tumor, CD8^+^ T cell population and activity increased in the combination therapy group (Figure 4G-H), and the T cell exhaustion markers PD-1 and Tim3 were decreased in the same group (Figure 4I-J). Also, the CD4^+^ T cell population increased, but the Tregs decreased in the combination therapy group compared to the vehicle group (Figure 4K-L). Finally, we performed immunohistochemistry (IHC) to study the combined effect of MAC and anti-PD-L1 on CD8^+^ T cell infiltration. CD8^+^ T cell infiltration into the tumor was increased in the MAC or anti-PD-L1 group but significantly further increased in the combination therapy group (Figure 4M). These results show that the combination therapy of MAC and anti-PD-L1 systemically enhances antitumor immunity.

### Combination of MAC and anti-PD-L1 antibody reverses EV PD-L1-mediated immunosuppression

**Figure 5:**
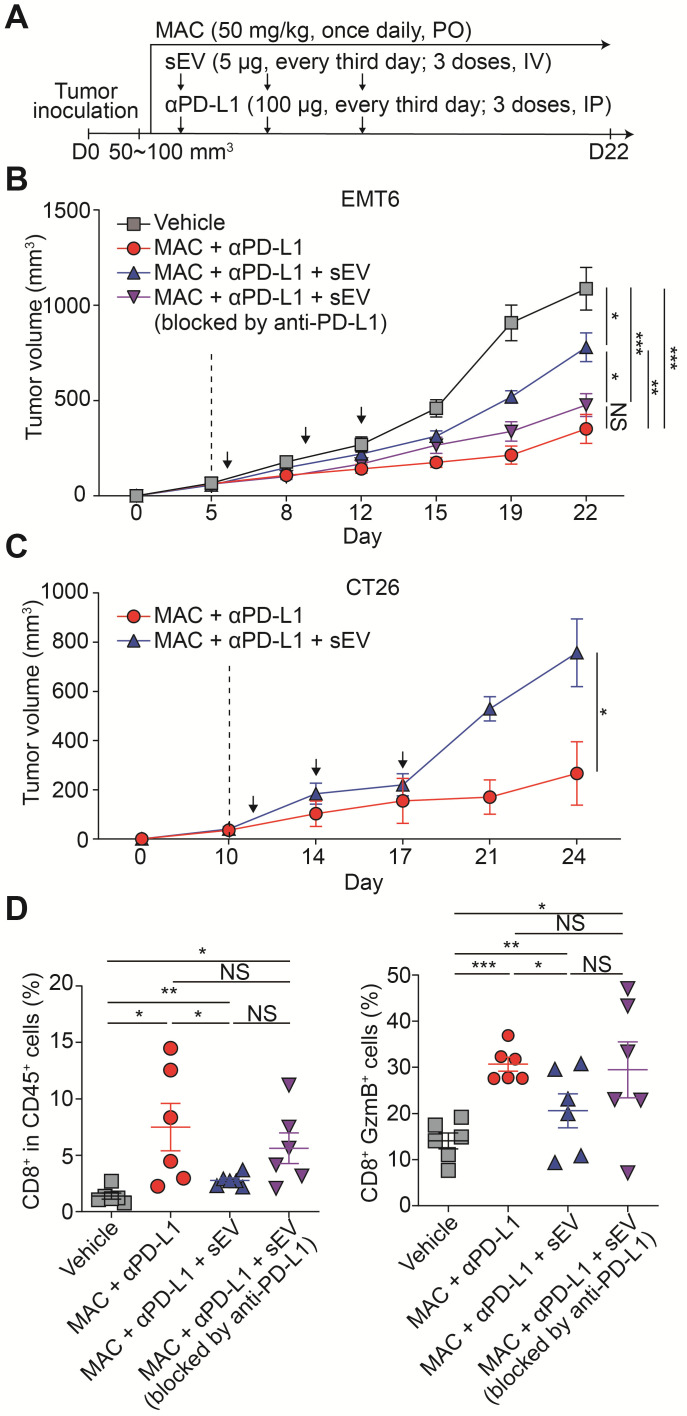
** Exogenous addition of EV PD-L1 reverses antitumor immunity induced by MAC and an anti-PD-L1 antibody. (A)** The design of the tumor regrowth experiment with EV PD-L1 injections in syngeneic cancer models. The growth curves of EMT6 **(B)** and CT26 **(C)** tumors in immunocompetent mice subjected to the indicated treatments. The vertical dotted lines indicate the beginning of MAC administration (at tumor volume = 50-100 mm^3^). **(D)** Flow cytometry analysis of the T lymphocytes from the EMT6 tumor-bearing mice with the indicated treatments, showing the proportion of CD8^+^ cells in CD45^+^ cells (left) and in the GzmB^+^ cells (right) in the tumor. The data are presented as means ± SEM (*n* = 6). *p < 0.05, **p < 0.01, ***p < 0.001, and ****p < 0.0001, respectively; NS, not significant; Mann-Whitney U test.

We studied whether the antitumor immunity boosted by the combination therapy could be reversed by injecting exogenous EV PD-L1. We injected EMT6 tumor-derived EVs intravenously into EMT6 tumor-bearing mice co-treated with MAC and the anti-PD-L1 antibody (Figure 5A). The EV injection restored tumor growth, whereas pretreating the EVs with anti-PD-L1 antibody did not achieve the restoration (Figure 5B, S10A). Similarly, in the MAC and anti-PD-L1 antibody-co-treated CT26 tumor-bearing mice, EV injection increased tumor growth (Figure 5C, S10B). In the EMT6 tumor-bearing mice, the population and cytotoxicity of the CD8^+^ T cells were decreased in the EV injection group (MAC + anti-PD-L1 + EV) more than the combination therapy group (MAC + anti-PD-L1), whereas injection with EVs blocked with anti-PD-L1 antibody (MAC + anti-PD-L1 + EV blocked by anti-PD-L1), displayed no significant changes (Figure 5D). These results show the role of EV PD-L1 in the synergistic effect of anti-PD-L1 and MAC.

### *EDNRA* expression is associated with EV secretion and immune responses

**Figure 6:**
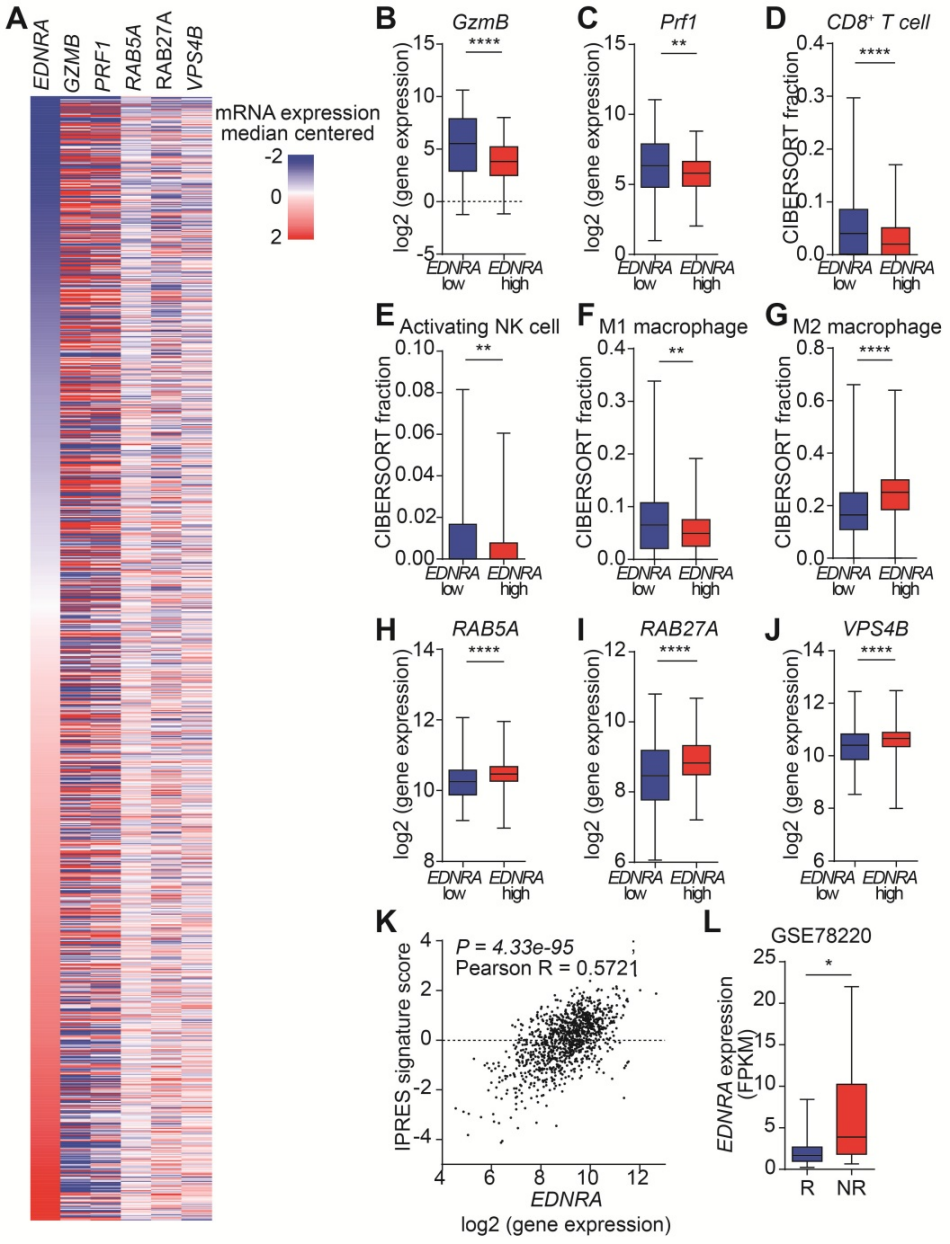
***EDNRA* expression is associated with immune response and EV secretion in breast cancer patients. (A)** Heatmap of immune and EV-related gene expression according to *EDNRA* gene expression in the 1082 breast cancer patients from the TCGA database. The gene expression levels of **(B)** *GzmB* and **(C)** *PRF1* according to *EDNRA* gene expression are shown. The correlation between the infiltration of **(D)** CD8^+^ T cells, **(E)** activated natural killer (NK) cells, **(F)** M1 macrophages, and **(G)** M2 macrophages was analyzed using CIBERSORT. The gene expression of **(H)** *RAB5A*, **(I)** *RAB27A*, and **(J)** *VPS4B* related to the *EDNRA* gene expression. **(K)** The correlation analysis between *EDNRA* expression and the IPRES gene signature. **(L)** The difference in *EDNRA* expression between the responders (*n* = 15) and non-responders (*n* = 13) to anti-PD-1 therapy from the Gene Expression Omnibus (GEO) database (GSE78220) of melanoma patient set. *p < 0.05, **p < 0.01, and ****p < 0.0001, respectively; Mann-Whitney U test.

The targeting of ETA by MAC increases the antitumor immunity in immunocompetent mice by inhibiting EVs. However, the effects of MAC on EV biogenesis/secretion and human immune responses are unknown. Therefore, we analyzed the clinical relevance of our findings using The Cancer Genome Atlas (TCGA) database with 1082 breast cancer patients (Figure 6A). First, we investigated the effect of* EDNRA* expression on tumor immunity. As expected, the expression of *GzmB* and *Prf1*, encoding GzmB and perforin, respectively, was high in the patients with low *EDNRA* expression (Figure 6B-C). Analysis of the immune cell population using CIBERSORT showed an increase of CD8^+^ T cells in the patients with low *EDNRA* expression, similar to the results in EMT6 bearing mice (Figure 6D). In addition, significantly increased natural killer (NK) cell activation and M1 macrophages with decreased M2 macrophages were observed in the patients with low *EDNRA* expression (Figure 6E-G).

Next, the changes in EV-related genes related to the expression of *EDNRA* were investigated. The genes associated with EV secretion, such as *RAB5A* and *RAB27A*, showed significantly lower expression in the low *EDNRA* expression patients than high *EDNRA* expression patients (Figure 6H-I). In addition, *VPS4B*, an important ESCRT-related regulatory component of intraluminal vesicles formation, decreased in the patients with low *EDNRA* expression (Figure 6J). These results are consistent with previous studies showing that ETA targeting affects EV biogenesis and secretion through the ESCRT-dependent mechanism 19. In addition, the patients with low *EDNRA* expression had higher disease-free survival rates (Figure S11).

We studied whether *EDNRA* expression was associated with PD-1 resistance. Thus, the correlation between innate PD-1 resistance signature (IPRES) and *EDNRA* expression was analyzed, and we observed a significantly positive correlation from TCGA data on breast cancer (Figure 6K, S11B). In addition, the Gene Expression Omnibus (GEO) database (GSE78220) of melanoma patients was used to evaluate the role of *EDNRA* in PD-1/PD-L1 blockade. Patients responsive to PD-1 therapy had lower *EDNRA* expression than the non-responders, suggesting that *EDNRA* was involved in the response to PD-1/PD-L1 blockade (Figure 6D). These results show that *EDNRA* expression is associated with EVs and immune responses in cancer patients and that using coadministration of MAC in PD-1/PD-L1 blockade therapy has clinical significance.

## Discussion

**Figure 7:**
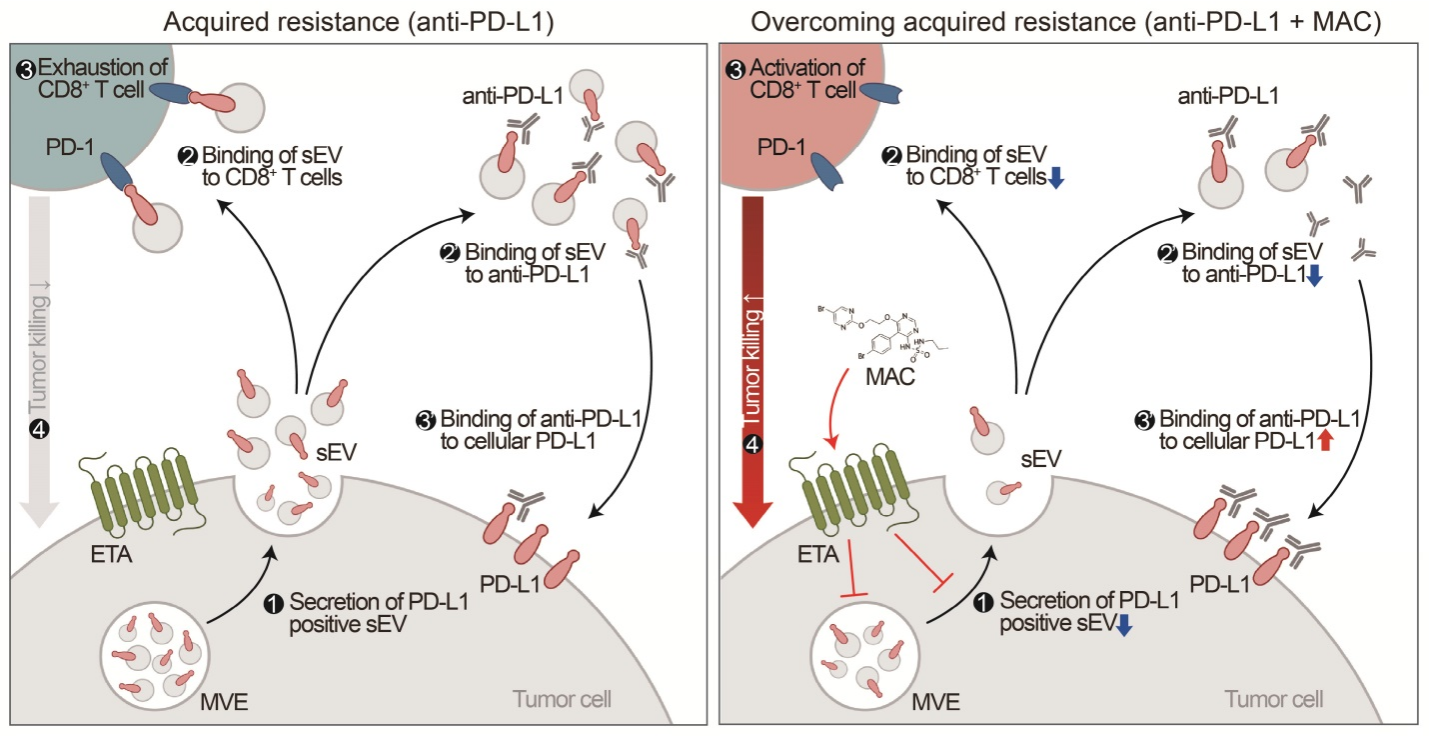
** Proposed model for MAC-mediated-enhanced cancer immunotherapy.** MAC increases the efficacy of the anti-PD-L1 antibody therapy and activates T cells by inhibiting EV PD-L1. ❶ First, MAC suppresses the release of EV PD-L1 from tumor cells by targeting ETA. ❷ The MAC-mediated suppression of EVs leads to reduced interaction of the remaining EVs with the PD-1 on CD8^+^ T cells or ❷′ with anti-PD-L1 antibodies. ❸′ Consequently, anti-PD-L1 antibodies, spared from binding to EV PD-L1, can bind to PD-L1 on the tumor cells, ❸ resulting in activation of CD8^+^ T cells. ❹ Ultimately, the MAC-mediated reinvigoration of exhausted CD8+T cells can cause tumor cell death and improve the overall anti-PD-L1 related ICB. MVE stands for multivesicular endosomes.

Although the chemical inhibitor GW4869 and the genetic suppression of EV PD-L1 can prevent EV PD-L1-mediated immunosuppression, corresponding FDA-approved drugs remain unavailable 15, 16, 29. Here, we have discovered that MAC, an FDA-approved drug, inhibits the secretion of tumor-derived EV PD-L1. Inhibition of EV PD-L1 secretion by ETA-targeting MAC reduces the binding of the EVs to the PD-1 on CD8^+^ T cells or anti-PD-L1 antibodies, reinvigorating the cytotoxicity of CD8^+^ T cells and thus enhancing antitumor immunity (Figure 7). The enhancement of anti-PD-L1 antibody efficacy by MAC, CD8^+^ T cell reinvigoration, and Treg cell reduction were confirmed in TNBC, colon, and lung cancer mouse xenograft models, indicating that MAC could be effective in various *EDNRA*-expressing cancer types. The analysis of clinical data revealed a positive correlation between high *EDNRA* expression and resistance to PD-1/PD-L1 blockade, suggesting that MAC could overcome the low response to ICB therapy. Our basic mechanistic and pre-translational study provides an improved treatment option for patients with a low response to PD-1/PD-L1 blockade, especially those with a high *EDNRA* expression and elevated levels of circulating EV PD-L1. Further evaluation of the clinical efficacy of MAC may be required.

EVs, including exosomes, play a role in cell proliferation and migration, angiogenesis, evasion of cell death, and invasion and metastasis in cancer 9, 10, 30. Tumor-derived EVs with PD-L1 on their surface are crucial and responsible for patients' low response to ICB 4, 12, 15, 16. In fact, EV PD-L1 are more stable than soluble PD-L1; they inhibit T cells more effectively because they simultaneously expresses MHC-1 31, 32. Moreover, EV PD-L1 transport PD-L1 to the breast cancer cells with low PD-L1 level 15, suggesting that suppressing EV PD-L1 not only enhances the efficacy of the anti-PD-L1 antibody but also impedes immune evasion by inhibiting PD-L1 transfer to other cells. Since it is necessary to discover new drugs that efficiently inhibit EV PD-L1 1, 5, 29, our findings have demonstrated the clinical value of an already-approved drug that enhances the efficacy of ICB by targeting a new mechanism.

As a strategy to suppress EV PD-L1, we considered suppression of the following three steps: 1) inhibition of PD-L1 on the surface of tumor cells, 2) impediment to PD-L1 sorting from tumor cells to EVs, and/or 3) inhibition of PD-L1-positive EV secretion. First, The PD-L1 of the EVs is derived from endosome formation by plasma membrane endocytosis 16, 33, 34. However, PD-L1 suppression on the tumor cell surface may pose unknown risks. For example, inhibiting cell surface PD-L1 increases the efficiency of anti-PD-1/PD-L1 therapy 35-38. In contrast, the efficacy of ICB is increased through the conversion from a cold tumor to a hot tumor by increasing PD-L1 39-42. Thus, the discrepancy in the results appears to depend on the types of patients receiving the specific treatment. Due to these contradictory results, it is difficult to demonstrate the increased antitumor immunity of reduced PD-L1-positive EVs by suppressing the PD-L1 on the tumor cell surface. Next, the mechanism by which elevated PD-L1 expression is sorted and secreted as exosomes has been studied. HRS, an ESCRT subunit required for transport, and ALIX, an ESCRT-related protein, have been shown to play a role in transporting PD-L1 on the tumor cell surface to EVs 4, 43. However, the detailed mechanisms how these proteins are directly involved in producing EVs need to be delineated. Finally, the immune response can be augmented by inhibiting PD-L1-positive EV secretion. Treatment with GW4869, a selective inhibitor of nSMase2, or knocking out *Smpd3* or *Rab27a* induces antitumor immunity by abolishing the secretion of PD-L1-positive EVs 15, 16. These results and our current results strongly indicate that blocking PD-L1-positive EV secretion has a great potential as a strategy to improve the patients' response to ICB therapy.

Recently, we have reported ETA's involvement in the secretion of EV 19. SFX, an oral antibacterial drug that targets ETA, inhibits EV biogenesis and secretion and triggers the colocalization of multivesicular endosomes (MVEs) with lysosomes for degradation in breast cancer cells, effectively blocking breast cancer growth and metastasis in xenograft models 19. This report showed that ETA regulates Rab proteins and ESCRT-related proteins related to EV secretion and biosynthesis and is involved in EV secretion by lysosomal degradation of MVE. Thus, the efficient antagonization of ETA is expect potently inhibit EV PD-L1. However, changes in the antibiotics-mediated gut microbiome are also associated with poor response to PD-1/PD-L1 blockade 44. Thus, it is necessary to screen for other drugs that antagonize ETA without significantly affecting the gut microbiota.

The endothelin axis, including ETA, is well established for potent vasoconstriction during the physiological regulation of vascular tone. Thus, the ETA axis is targeted to treat pulmonary arterial hypertension (PAH) 45, 46. MAC, identified via screening, is a dual ETA/ETB antagonist approved by the FDA for treating PAH. MAC is more effective with fewer side effects than bosentan 47, 48. In preclinical models, MAC exhibited sustained receptor binding and enhanced tissue penetration compared with other ETA antagonists, ambrisentan and bosentan 49. Unlike ambrisentan and bosentan, MAC is a noncompetitive antagonist with ET1 and ETA. It also has a long half-life and may effectively inhibit the secretion of EV PD-L1. In addition, since the ETA binding affinity of MAC (IC50s = 0.5 nM) is about 120 times higher than that of SFX (IC50s = 0.6 µM), MAC is likely to prevent EV PD-L1 efficiently at low concentrations 50, 51. In addition, MAC antagonizes ETA more specifically than ETB (ETA:ETB = 50:1) 50. In the current study, ETB antagonist BQ788 did not affect EV secretion. Further, the downregulation of Rab27a protein by MAC treatment is consistent with the results from the SFX and ETA KD model in the previous report 19. Therefore, this finding supports that the inhibition of EV PD-L1 by MAC is mediated mainly through ETA targeting and suppression. Therefore, the inhibition of EV PD-L1 by MAC is mediated mainly through ETA targeting and suppression.

ETA, associated with cardiovascular, inflammatory, fibrogenic and oncologic diseases, is involved in various physiological and pathological processes, such as vasoconstriction 45, 46, 52, 53. In particular, the binding of ET-1 and ETA in cancer induces the vascular endothelial growth factor expression by increasing levels of hypoxia-inducible factor 1α and consequently causes tumor angiogenesis 54-56. MAC with or without chemotherapy specifically reduces tumor growth in chemoresistant ovarian cancer 57, glioblastoma 58 and breast cancer 28, 59. All these previous studies suggest a direct tumor-killing effect by MAC. However, our current results demonstrate a novel mechanism for reinvigorating T cells by suppressing PD-L1 EV.

One study showed that endothelin B receptor mediates the endothelial barrier to T-cell homing to tumors and disables immune therapy, and ETB neutralization by BQ-788 increases T-cell homing to tumors 60. Targeting of ETB by MAC may be more effective in enhancing the efficacy of anti-PD-1/PD-L1 therapy due to increased T-cell infiltration. In addition, Son et al. reported that the polymeric nanoparticles bearing MAC prevent fibrotic progression by regulating the function of cancer-associated fibroblasts, attenuate the biogenesis of cancer cell-derived exosomes, and modulate the T cell subsets and distribution in TME, resulting effectively reorganize the immunosuppressive TME in the 4T1 tumor model. 61. These reports showed that antitumor immunity by MAC might be the result of multiple immunomodulating effects in TME and inhibition of EV PD-L1. Nevertheless, we clearly demonstrated the role of PD-L1 in EVs reduced by MAC through EV-mediated tumor rescues in multiple syngeneic models, and this results were interpreted that the reduction of EV PD-L1 by MAC significantly contribute to antitumor immunity.

In our results, the antitumor effect of MAC alone was observed in 4T1 and EMT6 models but not in CT26 and LL/2 tumor models. It is possible that the 50 mg/kg dose of MAC was not an effective concentration capable of showing antitumor effect in CT26 and LL/2 tumor models. We did not evaluate the antitumor effect according to the drug dose of each model in the *in vivo* study. Therefore, in future preclinical experiments, it is necessary to evaluate the effect of MAC using different doses in various tumor models. In addition, both 4T1 and EMT6 cells showed higher EV PD-L1 protein expression (Figure S4A) regulated by MAC compared with 4T1 and EMT6 cells, and this phenomenon might result in 4T1 and EMT6 cells being more susceptible to MAC. However, the combination with anti-PD-L1 showed a synergistic effect in various tumor models even if the variation in MAC alone for each animal model was observed. This suggested that the treatment of MAC alone with a dose of 50 mg/kg is insufficient for the antitumor effect in CT26 and LL/2 tumor models. However, MAC is still necessary for synergistic antitumor effect in the four tumor models for combination therapy.

Suppressing EV PD-L1 increases the number, cytotoxic activity and proliferation of CD8^+^ T cells and decreases their exhaustion in the tumors and DLNs in immunocompetent mice 4, 16. The current data of activation of the CD8^+^ T cells in tumors and DLNs in the EMT6 TNBC tumor model is consistent with previous reports. In addition, we report a reduction of Tregs in tumors and DLNs. *CD274*-expressing antigen-presenting cells contribute to the conversion of CD4^+^ T cells to Tregs by reducing Akt-mTOR signaling 62. Moreover, tumor-derived EVs can inhibit dendritic cell (DC) maturation, increasing Tregs 63. These reports suggest that EV PD-L1 can regulate the conversion of Tregs while MAC prevents this maturation process toward increased Treg population in our study. The advantage of immunotherapy is that immune cell activation induces long-term memory immunity. In the current study, we also confirmed that memory CD8^+^ T cells increased by MAC and anti-PD-L1 therapy to efficiently combat against tumor rechallenge. These results are consistent with the recent report that the inhibition of EV PD-L1 enhances long-term antitumor memory 16.

The TCGA database shows that *EDNRA* expression in breast cancer patients is associated with immune response and EV biogenesis and secretion. Tumor-derived EVs are involved in the immune response through PD-L1 and other mechanisms 64. Although our results showed the major effect of PD-L1 on EVs *in vitro*, it is difficult to exclude the effects of other EV components on immune cells *in vivo*. Tumor-derived EVs, including TRAIL and Fas ligand, induce the apoptosis of activated anti‐tumor T cells 65-67. In addition, breast cancer-derived EVs, including transforming growth factor‐β, mediates the suppression of CD8^+^ T cells and the proliferation of Tregs 68, 69. Moreover, EVs carrying NKG2D ligands can reduce the cytotoxicity of NK and CD8^+^ T cells by downregulating their surface *KLRK1* expression 70, 71. Furthermore, tumor-derived EVs enriched with miRNAs, such as miR‐21‐3p, miR‐125b‐5p, miR‐181d‐5p, and miR‐1246, induce the polarization of M2 macrophages with a robust protumor phenotype 72, 73. These reports indicate that the suppression of EVs by MAC represents a complex immune response through the regulation of PD-L1 and other immune activity mediators.

While MAC may be a coadministration agent capable of overcoming the low response to ICB therapy, it is necessary to elucidate its molecular mechanism in greater detail. MAC suppresses EV PD-L1 by inhibiting EV secretion. However, it is unclear how MAC affects the EV secretion from other immune cells, including T cells. *EDNRA* is generally overexpressed in tumors. Our results demonstrate that MAC enhances the killing effect of CD8^+^ T cells; however, its mechanism requires further study. In addition, PD-L1 is expressed not only in tumor cells but also in other cell types, including macrophages, DCs, and myeloid-derived suppressor cells, contributing to immunosuppression 12, 74. EV PD-L1 derived from these cells may be involved in immune evasion. Thus, the effect of EV PD-L1 derived from other cell types on immunosuppression requires further research, and drugs may need to be developed to address this effect.

The combination therapy of PD-1/PD-L1 blockade is tested on multiple targets in many clinical trials 75. However, despite the known importance of EV PD-L1 in immune evasion, there have no clinically approved drugs targeting them. Here, we have demonstrated the important value of MAC as a drug candidate that can overcome the immunosuppression mediated by tumor-derived EV PD-L1. Thus, MAC has significant clinical implications as a coadministration agent with PD-1/PD-L1 blockers.

## Supplementary Material

Supplementary figures.

## Abbreviations

sEV: Small extracellular vesicle
PD-L1: Programmed death-ligand 1
PD-1: Programmed death 1
MAC: Macitentan
ETA: Endothelin receptor A
TNBC: triple-negative breast cancer
ICB: Immune checkpoint blockade
TME: Tumor microenvironments
K/D: Knockdown
nSMase: Neutral sphingomyelinase gene
FDA: Food and Drug Administration
FBS: Fetal bovine serum
PBMC: Peripheral blood mononuclear cell
IFN-γ: Interferon-γ
HRP: Horseradish peroxidase
ECL: Enhanced chemiluminescence
APC: Allophycocyanin
V-SSC: Violet laser for side scatter
NTA: Nanoparticle tracking analysis
PBS: Phosphate-buffered saline
FITC: Fluorescein isothiocyanate
PE: Phycoerythrin
IPRES: Innate anti-PD-1 resistance
ssGSEA: Single sample gene set enrichment analysis
GSVA: Gene set variation analysis
GEO: Gene expression omnibus
ETB: Endothelin receptor B
WCL: Whole cell lysates
SFX: Sulfisoxazole
GzmB: Granzyme B
DLN: Draining lymph node
Tregs: Regulatory T cells
IHC: Immunohistochemistry
TCGA: The Cancer Genome Atlas
NK cell: natural killer cell
MVE: multivesicular endosome
PAH: Pulmonary arterial hypertension
DC: dendritic cell

## References

[1] Yin Z, Yu M, Ma T, Zhang C, Huang S, Karimzadeh MR (2021). Mechanisms underlying low-clinical responses to PD-1/PD-L1 blocking antibodies in immunotherapy of cancer: a key role of exosomal PD-L1. J Immunother Cancer.

[2] Mandal R, Samstein RM, Lee KW, Havel JJ, Wang H, Krishna C (2019). Genetic diversity of tumors with mismatch repair deficiency influences anti-PD-1 immunotherapy response. Science.

[3] Ren D, Hua Y, Yu B, Ye X, He Z, Li C (2020). Predictive biomarkers and mechanisms underlying resistance to PD1/PD-L1 blockade cancer immunotherapy. Mol Cancer.

[4] Chen G, Huang AC, Zhang W, Zhang G, Wu M, Xu W (2018). Exosomal PD-L1 contributes to immunosuppression and is associated with anti-PD-1 response. Nature.

[5] Xu Z, Zeng S, Gong Z, Yan Y (2020). Exosome-based immunotherapy: a promising approach for cancer treatment. Mol Cancer.

[6] Xie F, Xu M, Lu J, Mao L, Wang S (2019). The role of exosomal PD-L1 in tumor progression and immunotherapy. Mol Cancer.

[7] Tkach M, Thery C (2016). Communication by Extracellular Vesicles: Where We Are and Where We Need to Go. Cell.

[8] Bobrie A, Colombo M, Raposo G, Thery C (2011). Exosome secretion: molecular mechanisms and roles in immune responses. Traffic.

[9] Kalluri R, LeBleu VS (2020). The biology, function, and biomedical applications of exosomes. Science.

[10] Moller A, Lobb RJ (2020). The evolving translational potential of small extracellular vesicles in cancer. Nat Rev Cancer.

[11] McAndrews KM, Kalluri R (2019). Mechanisms associated with biogenesis of exosomes in cancer. Mol Cancer.

[12] Ricklefs FL, Alayo Q, Krenzlin H, Mahmoud AB, Speranza MC, Nakashima H (2018). Immune evasion mediated by PD-L1 on glioblastoma-derived extracellular vesicles. Sci Adv.

[13] Li M, Soder R, Abhyankar S, Abdelhakim H, Braun MW, Trinidad CV (2021). WJMSC-derived small extracellular vesicle enhance T cell suppression through PD-L1. J Extracell Vesicles.

[14] Su D, Tsai HI, Xu Z, Yan F, Wu Y, Xiao Y (2019). Exosomal PD-L1 functions as an immunosuppressant to promote wound healing. J Extracell Vesicles.

[15] Yang Y, Li CW, Chan LC, Wei Y, Hsu JM, Xia W (2018). Exosomal PD-L1 harbors active defense function to suppress T cell killing of breast cancer cells and promote tumor growth. Cell Res.

[16] Poggio M, Hu T, Pai CC, Chu B, Belair CD, Chang A (2019). Suppression of Exosomal PD-L1 Induces Systemic Anti-tumor Immunity and Memory. Cell.

[17] Li C, Li C, Zhi C, Liang W, Wang X, Chen X (2019). Clinical significance of PD-L1 expression in serum-derived exosomes in NSCLC patients. J Transl Med.

[18] Theodoraki MN, Yerneni SS, Hoffmann TK, Gooding WE, Whiteside TL (2018). Clinical Significance of PD-L1(+) Exosomes in Plasma of Head and Neck Cancer Patients. Clin Cancer Res.

[19] Im EJ, Lee CH, Moon PG, Rangaswamy GG, Lee B, Lee JM (2019). Sulfisoxazole inhibits the secretion of small extracellular vesicles by targeting the endothelin receptor A. Nat Commun.

[20] Thompson CA (2013). Macitentan approved by FDA to delay progression of PAH. Am J Health Syst Pharm.

[21] Thery C, Amigorena S, Raposo G, Clayton A (2006). Isolation and characterization of exosomes from cell culture supernatants and biological fluids. Curr Protoc Cell Biol.

[22] Choi D, Montermini L, Jeong H, Sharma S, Meehan B, Rak J (2019). Mapping Subpopulations of Cancer Cell-Derived Extracellular Vesicles and Particles by Nano-Flow Cytometry. ACS Nano.

[23] Hugo W, Zaretsky JM, Sun L, Song C, Moreno BH, Hu-Lieskovan S (2017). Genomic and Transcriptomic Features of Response to Anti-PD-1 Therapy in Metastatic Melanoma. Cell.

[24] Kim SJ, Kim JS, Kim SW, Yun SJ, He J, Brantley E (2012). Antivascular therapy for multidrug-resistant ovarian tumors by macitentan, a dual endothelin receptor antagonist. Transl Oncol.

[25] Kim SJ, Kim JS, Kim SW, Brantley E, Yun SJ, He J (2011). Macitentan (ACT-064992), a tissue-targeting endothelin receptor antagonist, enhances therapeutic efficacy of paclitaxel by modulating survival pathways in orthotopic models of metastatic human ovarian cancer. Neoplasia.

[26] Iglarz M, Landskroner K, Bauer Y, Vercauteren M, Rey M, Renault B (2015). Comparison of Macitentan and Bosentan on Right Ventricular Remodeling in a Rat Model of Non-vasoreactive Pulmonary Hypertension. J Cardiovasc Pharmacol.

[27] Schafer A, Haenig B, Erupathil J, Strickner P, Sabato D, Welford RWD (2021). Inhibition of endothelin-B receptor signaling synergizes with MAPK pathway inhibitors in BRAF mutated melanoma. Oncogene.

[28] Askoxylakis V, Ferraro GB, Badeaux M, Kodack DP, Kirst I, Shankaraiah RC (2019). Dual endothelin receptor inhibition enhances T-DM1 efficacy in brain metastases from HER2-positive breast cancer. NPJ Breast Cancer.

[29] Tang Y, Zhang P, Wang Y, Wang J, Su M, Wang Y (2020). The Biogenesis, Biology, and Clinical Significance of Exosomal PD-L1 in Cancer. Front Immunol.

[30] Hoshino A, Costa-Silva B, Shen TL, Rodrigues G, Hashimoto A, Tesic Mark M (2015). Tumour exosome integrins determine organotropic metastasis. Nature.

[31] Fan Y, Che X, Qu J, Hou K, Wen T, Li Z (2019). Exosomal PD-L1 Retains Immunosuppressive Activity and is Associated with Gastric Cancer Prognosis. Ann Surg Oncol.

[32] Cordonnier M, Nardin C, Chanteloup G, Derangere V, Algros MP, Arnould L (2020). Tracking the evolution of circulating exosomal-PD-L1 to monitor melanoma patients. J Extracell Vesicles.

[33] Mezzadra R, Sun C, Jae LT, Gomez-Eerland R, de Vries E, Wu W (2017). Identification of CMTM6 and CMTM4 as PD-L1 protein regulators. Nature.

[34] Burr ML, Sparbier CE, Chan YC, Williamson JC, Woods K, Beavis PA (2017). CMTM6 maintains the expression of PD-L1 and regulates anti-tumour immunity. Nature.

[35] Luo F, Luo M, Rong QX, Zhang H, Chen Z, Wang F (2019). Niclosamide, an antihelmintic drug, enhances efficacy of PD-1/PD-L1 immune checkpoint blockade in non-small cell lung cancer. J Immunother Cancer.

[36] Wu RY, Kong PF, Xia LP, Huang Y, Li ZL, Tang YY (2019). Regorafenib Promotes Antitumor Immunity via Inhibiting PD-L1 and IDO1 Expression in Melanoma. Clin Cancer Res.

[37] Ye Y, Kuang X, Xie Z, Liang L, Zhang Z, Zhang Y (2020). Small-molecule MMP2/MMP9 inhibitor SB-3CT modulates tumor immune surveillance by regulating PD-L1. Genome Med.

[38] Vannini A, Leoni V, Barboni C, Sanapo M, Zaghini A, Malatesta P (2019). alphavbeta3-integrin regulates PD-L1 expression and is involved in cancer immune evasion. Proc Natl Acad Sci U S A.

[39] Noman MZ, Parpal S, Van Moer K, Xiao M, Yu Y, Viklund J (2020). Inhibition of Vps34 reprograms cold into hot inflamed tumors and improves anti-PD-1/PD-L1 immunotherapy. Sci Adv.

[40] Tu MM, Lee FYF, Jones RT, Kimball AK, Saravia E, Graziano RF (2019). Targeting DDR2 enhances tumor response to anti-PD-1 immunotherapy. Sci Adv.

[41] Sen T, Della Corte CM, Milutinovic S, Cardnell RJ, Diao L, Ramkumar K (2019). Combination Treatment of the Oral CHK1 Inhibitor, SRA737, and Low-Dose Gemcitabine Enhances the Effect of Programmed Death Ligand 1 Blockade by Modulating the Immune Microenvironment in SCLC. J Thorac Oncol.

[42] Sen T, Rodriguez BL, Chen L, Corte CMD, Morikawa N, Fujimoto J (2019). Targeting DNA Damage Response Promotes Antitumor Immunity through STING-Mediated T-cell Activation in Small Cell Lung Cancer. Cancer Discov.

[43] Monypenny J, Milewicz H, Flores-Borja F, Weitsman G, Cheung A, Chowdhury R (2018). ALIX Regulates Tumor-Mediated Immunosuppression by Controlling EGFR Activity and PD-L1 Presentation. Cell Rep.

[44] Routy B, Le Chatelier E, Derosa L, Duong CPM, Alou MT, Daillere R (2018). Gut microbiome influences efficacy of PD-1-based immunotherapy against epithelial tumors. Science.

[45] Enevoldsen FC, Sahana J, Wehland M, Grimm D, Infanger M, Kruger M (2020). Endothelin Receptor Antagonists: Status Quo and Future Perspectives for Targeted Therapy. J Clin Med.

[46] Aubert JD, Juillerat-Jeanneret L (2016). Endothelin-Receptor Antagonists beyond Pulmonary Arterial Hypertension: Cancer and Fibrosis. J Med Chem.

[47] Bolli MH, Boss C, Binkert C, Buchmann S, Bur D, Hess P (2012). The discovery of N-[5-(4-bromophenyl)-6-[2-[(5-bromo-2-pyrimidinyl)oxy]ethoxy]-4-pyrimidinyl]-N'-p ropylsulfamide (Macitentan), an orally active, potent dual endothelin receptor antagonist. J Med Chem.

[48] Bedan M, Grimm D, Wehland M, Simonsen U, Infanger M, Kruger M (2018). A Focus on Macitentan in the Treatment of Pulmonary Arterial Hypertension. Basic Clin Pharmacol Toxicol.

[49] Gatfield J, Mueller Grandjean C, Sasse T, Clozel M, Nayler O (2012). Slow receptor dissociation kinetics differentiate macitentan from other endothelin receptor antagonists in pulmonary arterial smooth muscle cells. PLoS One.

[50] Iglarz M, Binkert C, Morrison K, Fischli W, Gatfield J, Treiber A (2008). Pharmacology of macitentan, an orally active tissue-targeting dual endothelin receptor antagonist. J Pharmacol Exp Ther.

[51] Chan MF, Okun I, Stavros FL, Hwang E, Wolff ME, Balaji VN (1994). Identification of a new class of ETA selective endothelin antagonists by pharmacophore directed screening. Biochem Biophys Res Commun.

[52] Davenport AP, Hyndman KA, Dhaun N, Southan C, Kohan DE, Pollock JS (2016). Endothelin. Pharmacol Rev.

[53] Barton M, Yanagisawa M (2019). Endothelin: 30 Years From Discovery to Therapy. Hypertension.

[54] Knowles J, Loizidou M, Taylor I (2005). Endothelin-1 and angiogenesis in cancer. Curr Vasc Pharmacol.

[55] Smollich M, Gotte M, Kersting C, Fischgrabe J, Kiesel L, Wulfing P (2008). Selective ETAR antagonist atrasentan inhibits hypoxia-induced breast cancer cell invasion. Breast Cancer Res Treat.

[56] Harris AL (2002). Hypoxia-a key regulatory factor in tumour growth. Nat Rev Cancer.

[57] Sestito R, Cianfrocca R, Rosano L, Tocci P, Di Castro V, Caprara V (2016). Macitentan blocks endothelin-1 receptor activation required for chemoresistant ovarian cancer cell plasticity and metastasis. Life Sci.

[58] Kim SJ, Lee HJ, Kim MS, Choi HJ, He J, Wu Q (2015). Macitentan, a Dual Endothelin Receptor Antagonist, in Combination with Temozolomide Leads to Glioblastoma Regression and Long-term Survival in Mice. Clin Cancer Res.

[59] Lee HJ, Hanibuchi M, Kim SJ, Yu H, Kim MS, He J (2016). Treatment of experimental human breast cancer and lung cancer brain metastases in mice by macitentan, a dual antagonist of endothelin receptors, combined with paclitaxel. Neuro Oncol.

[60] Buckanovich RJ, Facciabene A, Kim S, Benencia F, Sasaroli D, Balint K (2008). Endothelin B receptor mediates the endothelial barrier to T cell homing to tumors and disables immune therapy. Nat Med.

[61] Son S, Shin JM, Shin S, Kim CH, Lee JA, Ko H (2021). Repurposing macitentan with nanoparticle modulates tumor microenvironment to potentiate immune checkpoint blockade. Biomaterials.

[62] Francisco LM, Salinas VH, Brown KE, Vanguri VK, Freeman GJ, Kuchroo VK (2009). PD-L1 regulates the development, maintenance, and function of induced regulatory T cells. J Exp Med.

[63] Ning Y, Shen K, Wu Q, Sun X, Bai Y, Xie Y (2018). Tumor exosomes block dendritic cells maturation to decrease the T cell immune response. Immunol Lett.

[64] Xie F, Zhou X, Fang M, Li H, Su P, Tu Y (2019). Extracellular Vesicles in Cancer Immune Microenvironment and Cancer Immunotherapy. Adv Sci (Weinh).

[65] Peng P, Yan Y, Keng S (2011). Exosomes in the ascites of ovarian cancer patients: origin and effects on anti-tumor immunity. Oncol Rep.

[66] Abusamra AJ, Zhong Z, Zheng X, Li M, Ichim TE, Chin JL (2005). Tumor exosomes expressing Fas ligand mediate CD8+ T-cell apoptosis. Blood Cells Mol Dis.

[67] Reichmann E (2002). The biological role of the Fas/FasL system during tumor formation and progression. Semin Cancer Biol.

[68] Liu Y, Zhang P, Li J, Kulkarni AB, Perruche S, Chen W (2008). A critical function for TGF-beta signaling in the development of natural CD4+CD25+Foxp3+ regulatory T cells. Nat Immunol.

[69] Rong L, Li R, Li S, Luo R (2016). Immunosuppression of breast cancer cells mediated by transforming growth factor-beta in exosomes from cancer cells. Oncol Lett.

[70] Lundholm M, Schroder M, Nagaeva O, Baranov V, Widmark A, Mincheva-Nilsson L (2014). Prostate tumor-derived exosomes down-regulate NKG2D expression on natural killer cells and CD8+ T cells: mechanism of immune evasion. PLoS One.

[71] Clayton A, Mitchell JP, Court J, Linnane S, Mason MD, Tabi Z (2008). Human tumor-derived exosomes down-modulate NKG2D expression. J Immunol.

[72] Chen X, Zhou J, Li X, Wang X, Lin Y, Wang X (2018). Exosomes derived from hypoxic epithelial ovarian cancer cells deliver microRNAs to macrophages and elicit a tumor-promoted phenotype. Cancer Lett.

[73] Cooks T, Pateras IS, Jenkins LM, Patel KM, Robles AI, Morris J (2018). Mutant p53 cancers reprogram macrophages to tumor supporting macrophages via exosomal miR-1246. Nat Commun.

[74] Bloch O, Crane CA, Kaur R, Safaee M, Rutkowski MJ, Parsa AT (2013). Gliomas promote immunosuppression through induction of B7-H1 expression in tumor-associated macrophages. Clin Cancer Res.

[75] Topalian SL, Taube JM, Pardoll DM (2020). Neoadjuvant checkpoint blockade for cancer immunotherapy. Science.

