# Supplementary material for: Macitentan improves antitumor immune responses by inhibiting the secretion of tumor-derived extracellular vesicle PD-L1
Source: Theranostics. 2022 Jan 31;12(5):1971–87. doi: 10.7150/thno.68864 (PMC8899590; doi:10.7150/thno.68864)
Supplement: Supplementary file 1 — Supplementary figures. [file thnov12p1971s1.pdf]

Supplementary Material

Macitentan improves antitumor immune responses by inhibiting the secretion of tumor-derived extracellular vesicle PD-L1

Chan-Hyeong Lee[1], Ju-Hyun Bae[1], Eun-Ji Choe[1], Ju-Mi Park[1], Sung-Sik Park[1], Hee Jin Cho[2], Byoung-Joon Song[3] and Moon-Chang Baek[1]*

*Corresponding author. Email: mcbaek@knu.ac.kr

Supplementary Figure

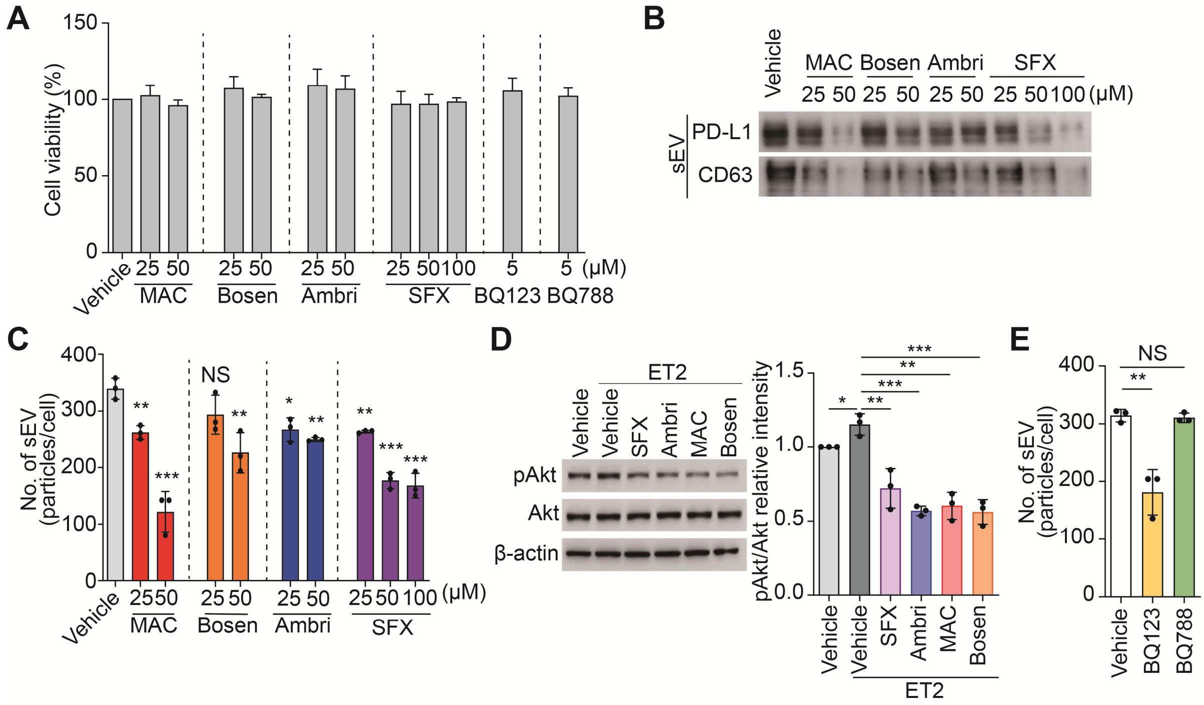

Figure S1. ETA antagonists suppress EV PD-L1 from MDA-MB231 cells. (A) The cell cytotoxicity induced by the ETA/ETB antagonists at the indicated concentrations. (B) The immunoblots of PD-L1 and CD63 in the EVs derived from the MDA-MB231 cells treated with ETA antagonists. (C) The number of the EVs derived from the MDA-MB231 cells treated with the indicated concentration of ETA antagonists was measured by nanoparticle tracking analysis. (D) The immunoblot of Akt and phosphorylated Akt in the MDA-MB231 cells treated with the indicated ET2 and ETA antagonists (SFX, ambrisentan, MAC and bosentan). The densitometric analysis of the relative intensity of the protein bands (right). (E) The number of the EVs derived from the MDA-MB231 cells treated with BQ123 or BQ788. The data are presented as means ± SD ($n = 3$). *$p < 0.05$; **$p < 0.01$; ***$p < 0.001$; NS, not significant.

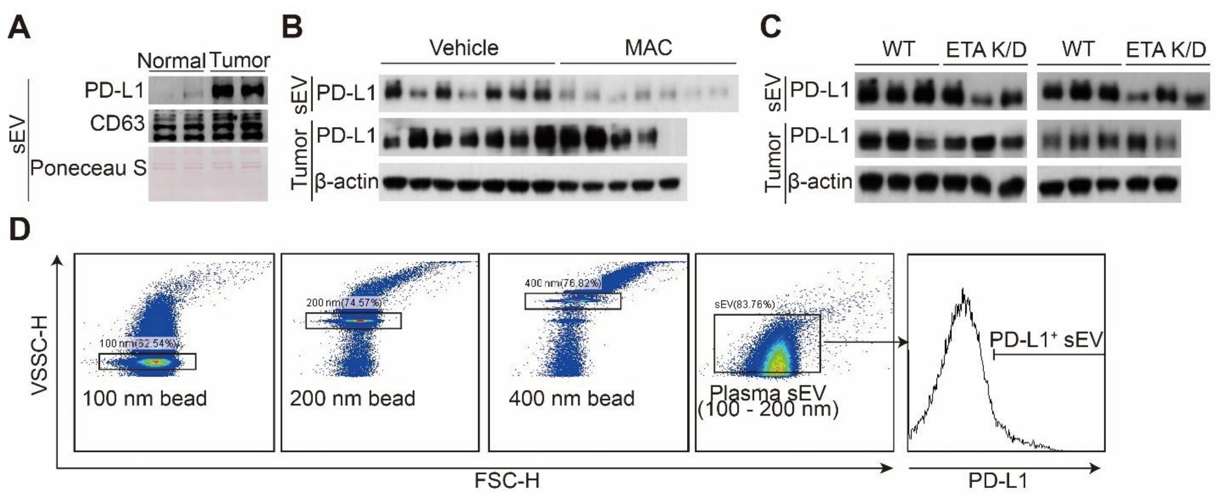

Figure S2. ETA disruption or MAC treatment inhibits circulating EV PD-L1 in the MDA-MB231 xenograft model. (A) The immunoblots of the PD-L1 in circulating EVs from mice with or without MDA-MB231 tumor. (B) The immunoblots of the PD-L1 in circulating EVs and tumor lysates from MDA-MB231 xenograft models in Figure 1K (left, n = 5 and 7, respectively) and (C) WT or ETA K/D in Figure 1L (left, n = 5 and 6, respectively). (D) Beads of different sizes, i.e., 100, 200, and 400 nm, were identified with V-SSC (describe its full-name). EV PD-L1 were measured on gates with a size of 100 to 200 nm, which is considered the EV population.

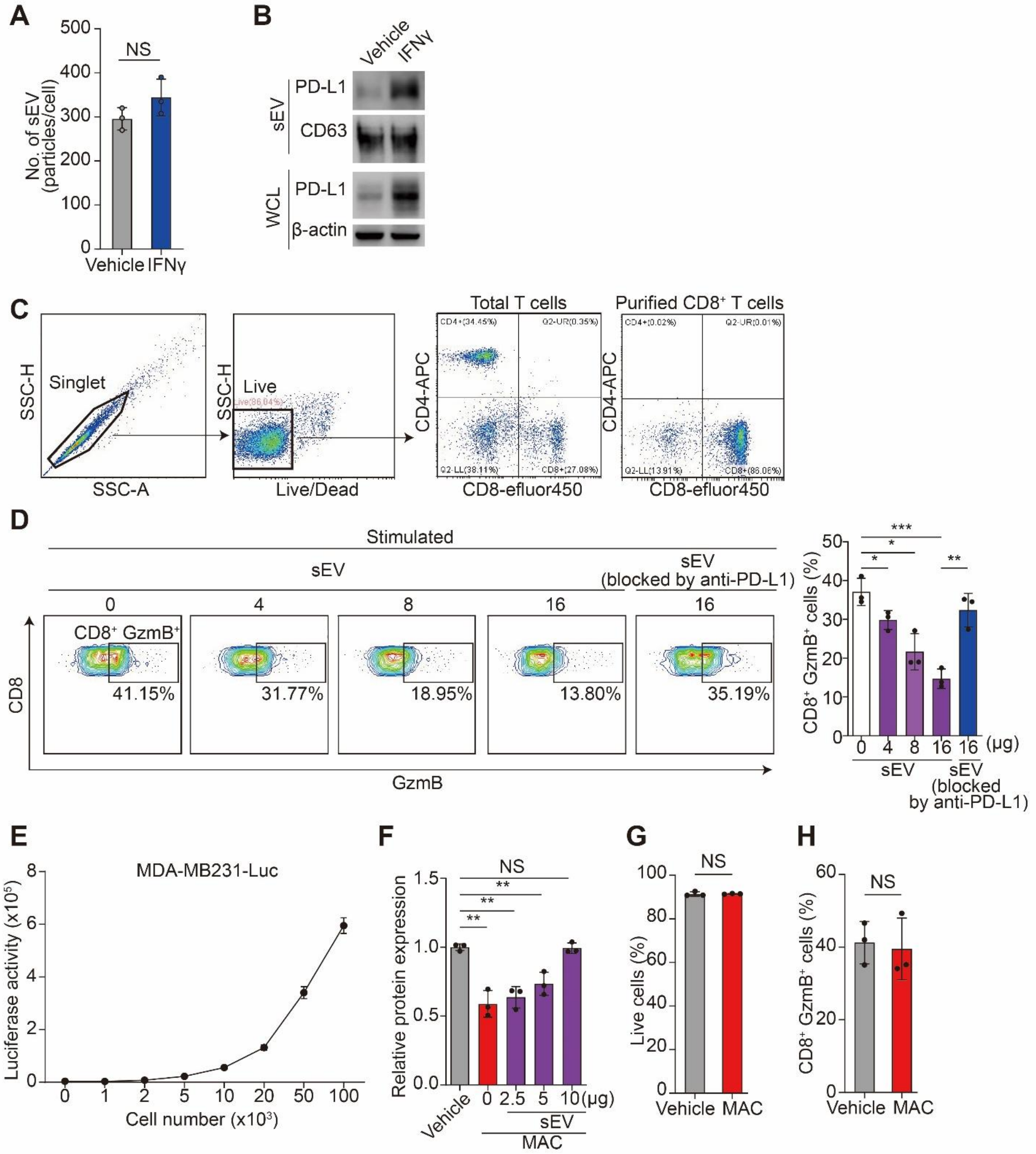

A
B
C
D
E
F
G
H
No. of sEV (particles/cell)
NS
Vehicle
IFNγ
sEV
WCL
PD-L1
CD63
β-actin
SSC-H
SSC-A
Singlet
Live
Live/Dead
Total T cells
Purified CD8⁺ T cells
CD4-APC
CD8-efluor450
Stimulated
sEV
sEV (blocked by anti-PD-L1)
0
4
8
16
CD8⁺ GzmB⁺
41.15%
31.77%
18.95%
13.80%
35.19%
CD8
GzmB
CD8⁺ GzmB⁺ cells (%)
(μg)
MDA-MB231-Luc
Luciferase activity (x10⁵)
Cell number (x10³)
Relative protein expression
MAC
Live cells (%)

Figure S3. EV PD-L1 regulate CD8$^+$ T cell activity. (A) The number of EVs derived from MDA-MB231 cells with or without interferon-γ (IFNγ). (B) The immunoblot of various proteins in EVs and whole-cell lysates (WCL) from the MDA-MB231 cells with or without IFN-γ treatment. (C) A gating strategy was used to identify the purified CD8$^+$ T cells from human PBMCs. (D) The cytotoxicity activities of human CD8$^+$ T cells treated with different concentrations of EV PD-L1 derived from MDA-MB231 cells or blocked by anti-PD-L1 were examined for granzyme B (GzmB) levels (left), showing the proportions of the CD8$^+$ T cells with activated GzmB levels (right). (E) The luciferase activity of MDA-MB231-luciferase cells with different cell numbers. (F) Human CD8$^+$ T cell-mediated cytotoxicity assay in MDA-MB231-luciferase cells with different concentrations of EVs under the treatment of MAC. (G) The cell viability of and (H) GzmB level in the CD8$^+$ T cells with or without MAC. The data are presented as means ± SD (n = 3). *p < 0.05, **p < 0.01, ***p < 0.001, respectively; NS, not significant.

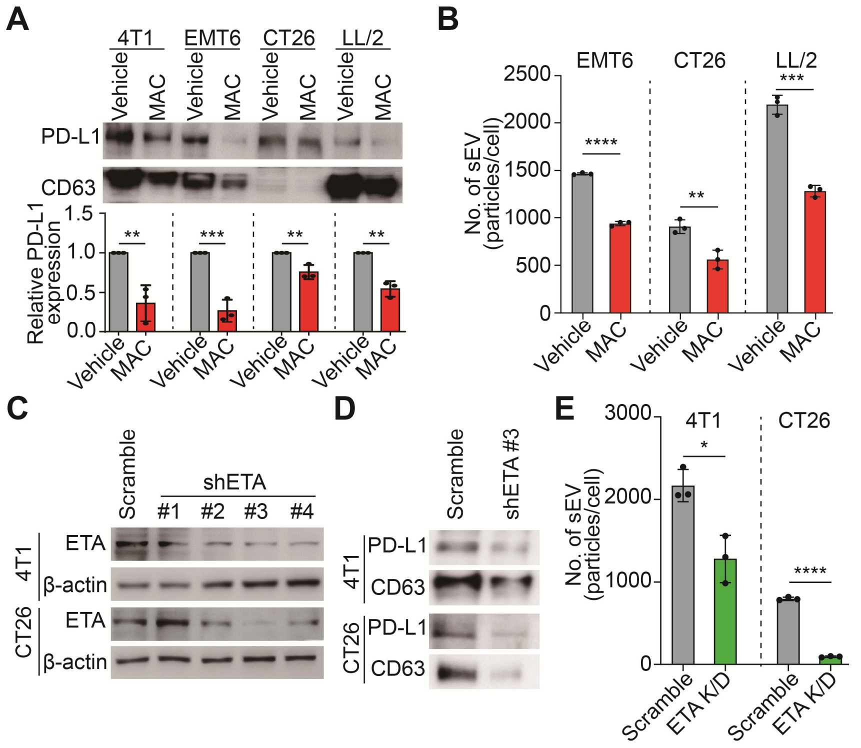

Figure S4. MAC suppresses EV PD-L1 from various mouse cancer cells. (A) The immunoblot of PD-L1 and CD63 in the EVs from 4T1, EMT6, CT26 and LL/2 mouse cancer cells with or without MAC treatment. The EV proteins from 1×10^7 cells were loaded per lane. (B) The number of the EVs derived from mouse cancer cells with or without MAC. (C) The immunoblot of ETA from ETA K/D 4T1 and CT26 cells. Beta-actin was used as the loading control. (D) The immunoblot of PD-L1 and CD63 in the EVs from ETA K/D 4T1 and CT26 cells. (E) The number of the EVs derived from ETA K/D 4T1 and CT26 cells. The data are presented as means ± SD ($n = 3$). *$p < 0.05$, **$p < 0.01$, ***$p < 0.001$, and ****$p < 0.0001$, respectively.

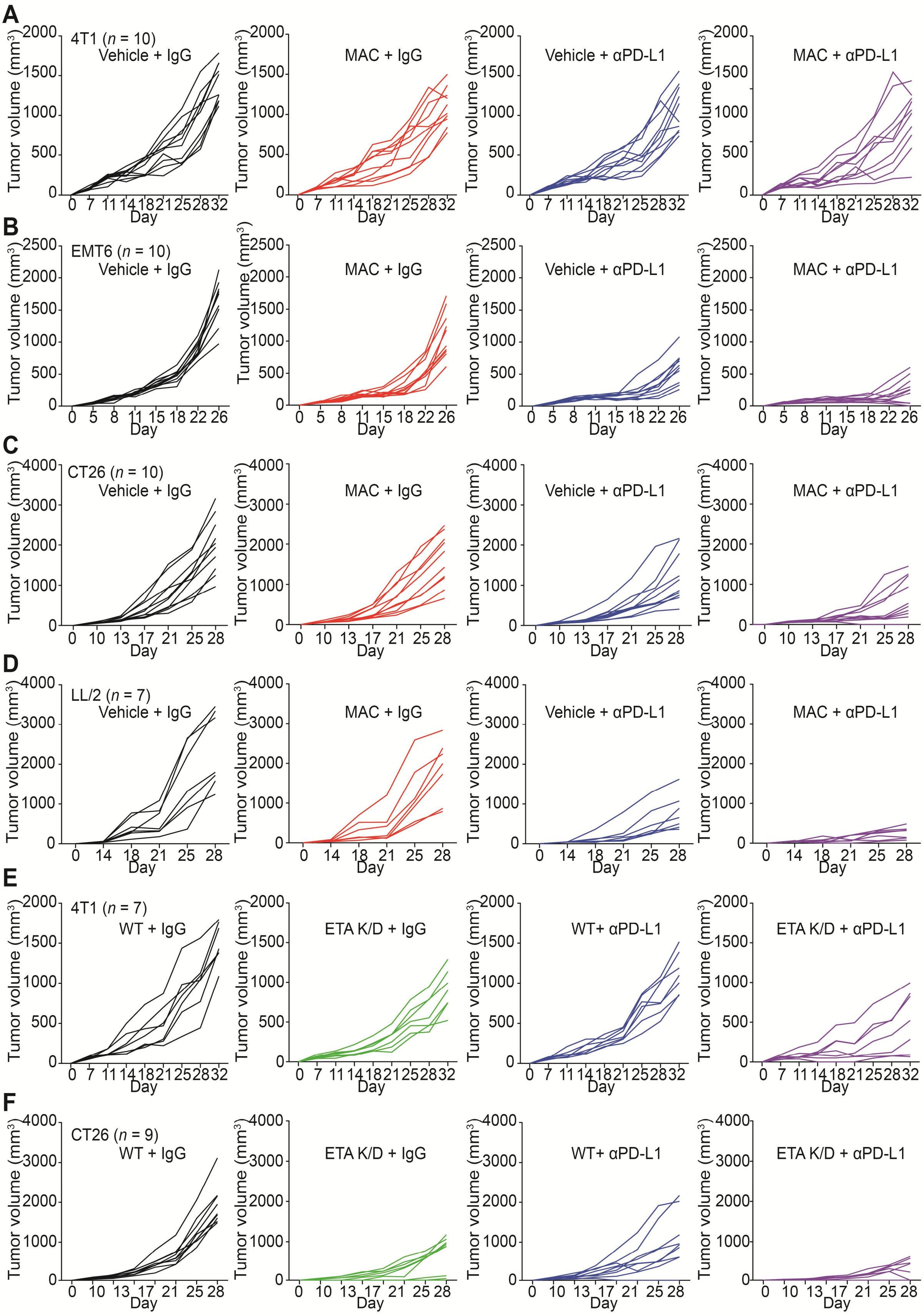
A
4T1 (n = 10)
Vehicle + IgG
MAC + IgG
Vehicle + αPD-L1
MAC + αPD-L1
B
EMT6 (n = 10)
Vehicle + IgG
MAC + IgG
Vehicle + αPD-L1
MAC + αPD-L1
C
CT26 (n = 10)
Vehicle + IgG
MAC + IgG
Vehicle + αPD-L1
MAC + αPD-L1
D
LL/2 (n = 7)
Vehicle + IgG
MAC + IgG
Vehicle + αPD-L1
MAC + αPD-L1
E
4T1 (n = 7)
WT + IgG
ETA K/D + IgG
WT+ αPD-L1
ETA K/D + αPD-L1
F
CT26 (n = 9)
WT + IgG
ETA K/D + IgG
WT+ αPD-L1
ETA K/D + αPD-L1
Tumor volume (mm³)
Day

Figure S5. MAC treatment or ETA disruption enhances the efficacy of anti-PD-L1 antibody therapy in mice. The growth curves of individual tumors in each treatment group of 4T1 (A), EMT6 (B), CT26 (C), and LL/2 (D). The growth curves of individual tumors in each group from 4T1 (E) and CT26 (F) tumor-bearing mice with ETA K/D group (n = 7 and 10, respectively).

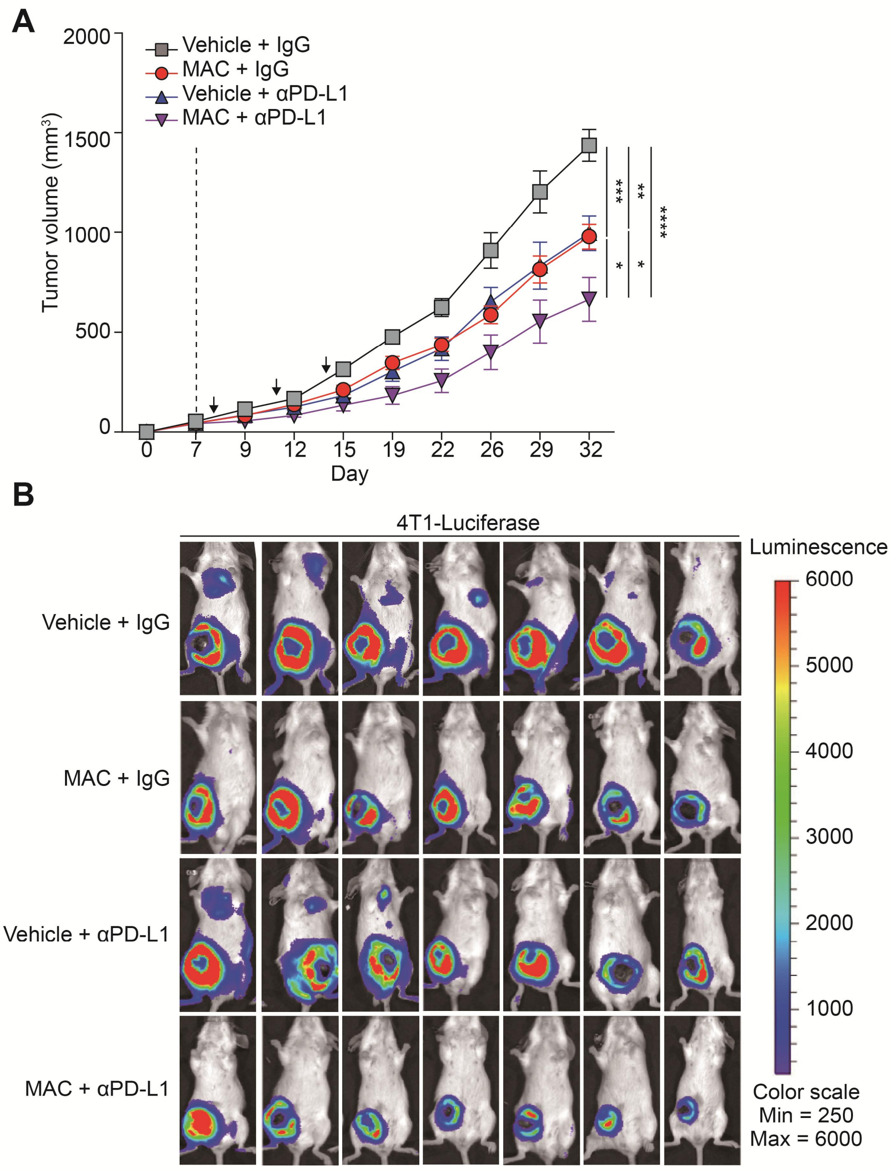
A
Vehicle + IgG
MAC + IgG
Vehicle + αPD-L1
MAC + αPD-L1
Tumor volume (mm3)
2000
1500
1000
500
0
0
7
9
12
15
19
22
26
29
32
Day
***
**
****
*
*
B
4T1-Luciferase
Luminescence
Vehicle + IgG
MAC + IgG
Vehicle + αPD-L1
MAC + αPD-L1
6000
5000
4000
3000
2000
1000
Color scale
Min = 250
Max = 6000

Figure S6. MAC inhibits lung metastasis in the 4T1 syngeneic model. (A) The growth curves of 4T1-luciferase tumors in immunocompetent mice treated with the vehicle or MAC with isotype IgG or anti-PD-L1 antibody. The vertical dotted line indicates the start date of drug administration (at tumor volume = 50–100 mm^3). Three blue arrows represent the timing of anti-PD-L1 antibody administration. (B) The cancer cells in the mice were tracked with the IVIS imaging system following the injection of 4T1-luciferase cells on day 32. The data are presented as means ± SEM ($n = 7$). $^{*}p < 0.05$, $^{**}p < 0.01$, $^{***}p < 0.001$, and $^{****}p < 0.0001$, respectively; Mann–Whitney U test.

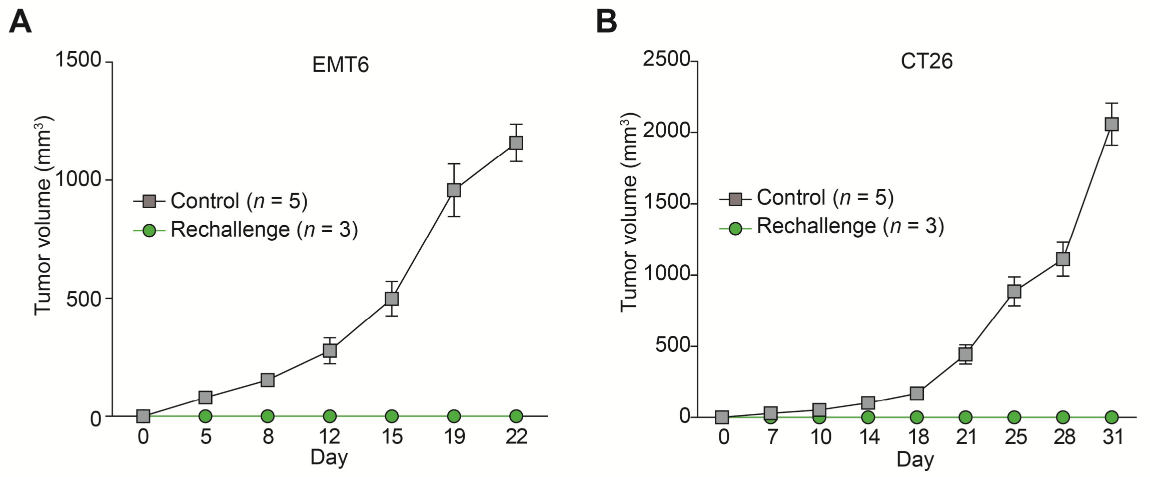

Figure S7. Combination of MAC and the anti-PD-L1 antibody enhanced the antitumor immune memory in the syngeneic cancer models. Changes in tumor volumes are shown after tumor rechallenge in the (A) EMT6 and (B) CT26 models. After treatment with MAC and anti-PD-L1 antibody, the complete tumor regression mice and age-matched controls were rechallenged with tumor cells (n = 3 and 5, respectively).

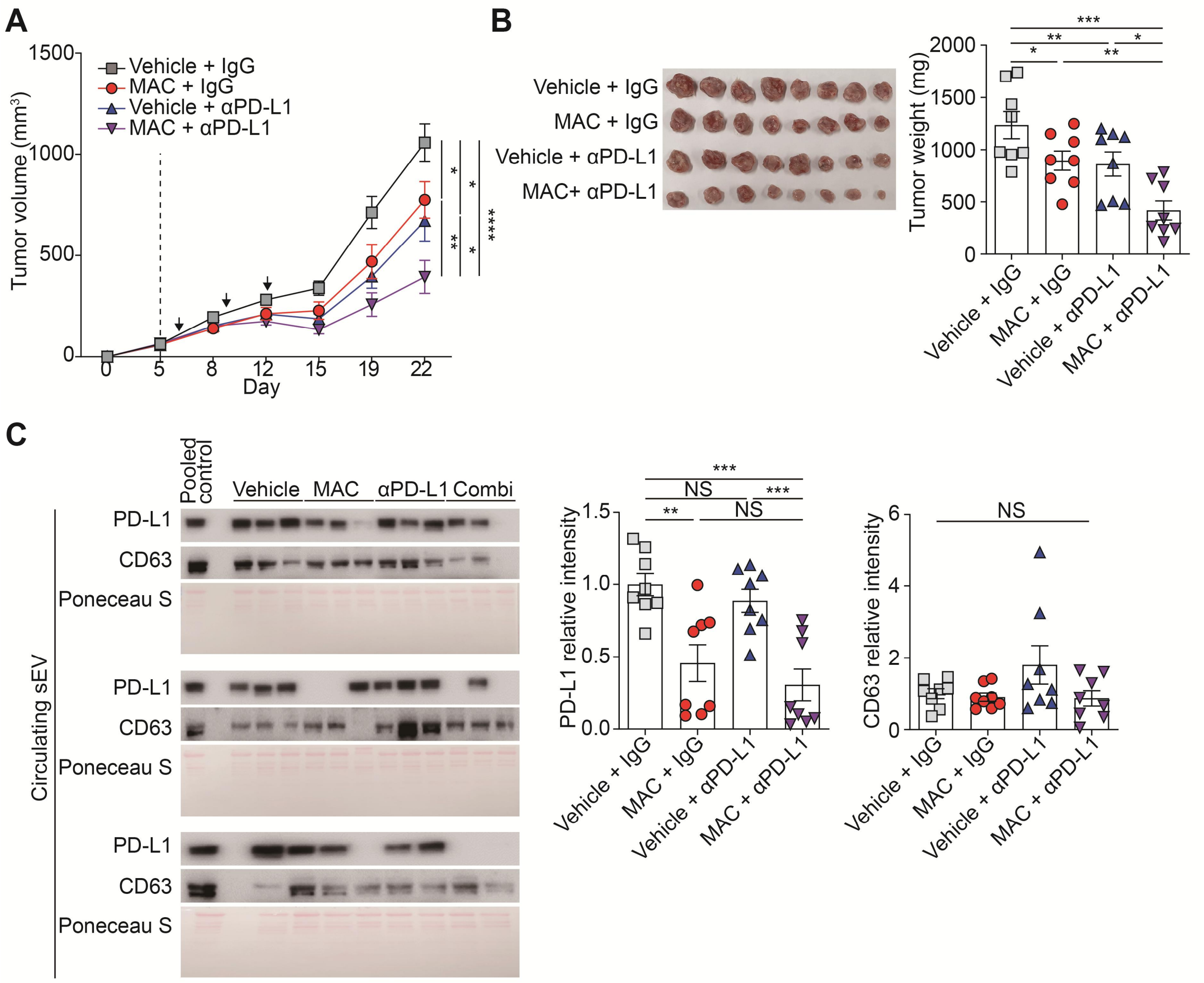

Figure S8. The combination therapy of MAC and anti-PD-L1 antibody inhibits tumor growth and EV PD-L1 in the EMT6 tumor model. (A) Tumor growth curves and (B) tumor weights of the EMT6 tumors in immunocompetent mice treated with the vehicle or MAC with isotype IgG or anti-PD-L1 antibody for immunophenotyping. (C) The immunoblot of PD-L1 of the circulating EVs treated with or without MAC (left). The densitometric analysis of the relative intensity of the protein bands (right). The data are presented as means ± SEM (n = 8). *$p < 0.05$, **$p < 0.01$, ***$p < 0.001$, and ****$p < 0.0001$, respectively; NS, not significant; Mann–Whitney U test.

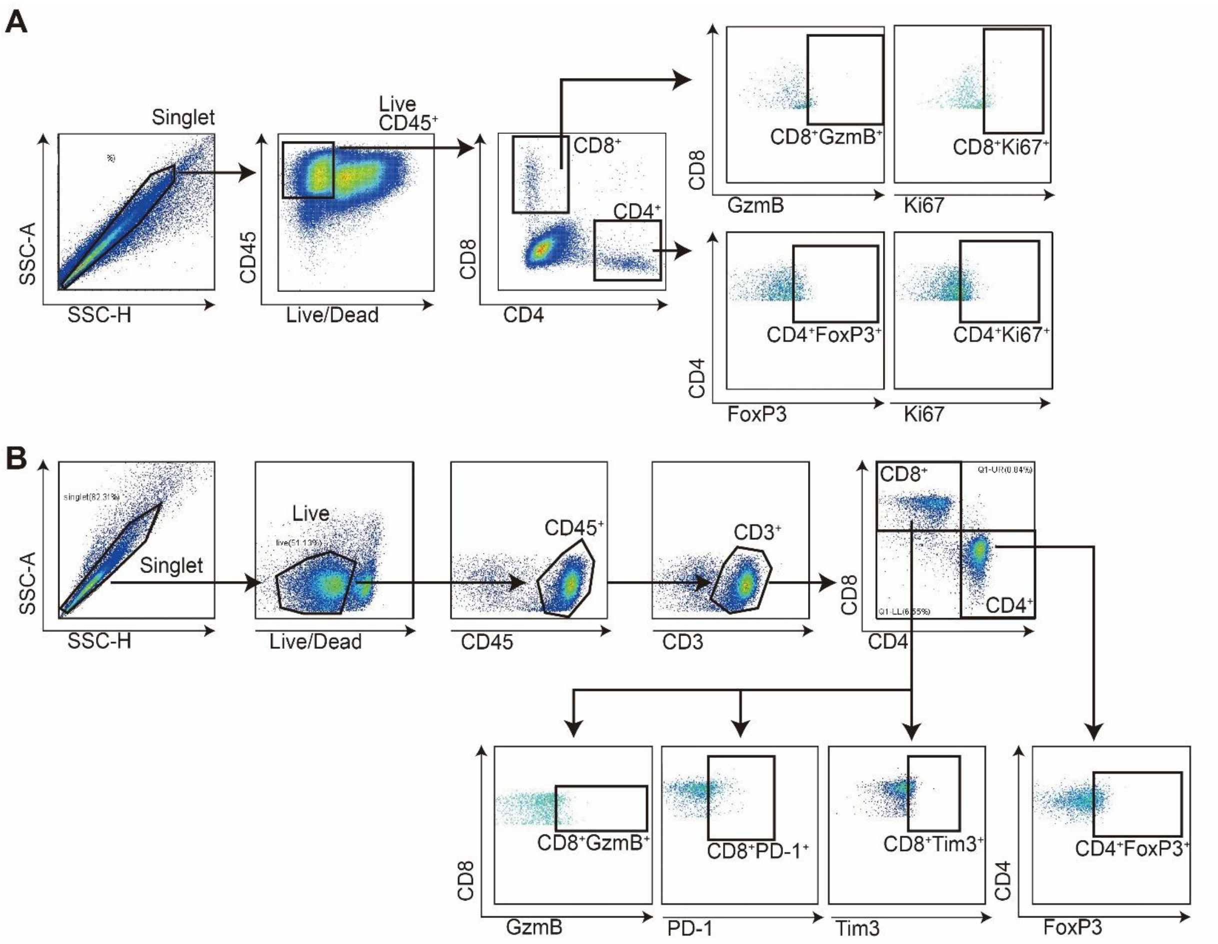

Figure S9. The gating strategy used in flow cytometry analysis. The gating strategy for immunophenotyping the cells in (A) tumor and (B) DLN. A time gate was initially applied to exclude any doublets, and dead cells, then all cells were categorized based on indicated antibodies.

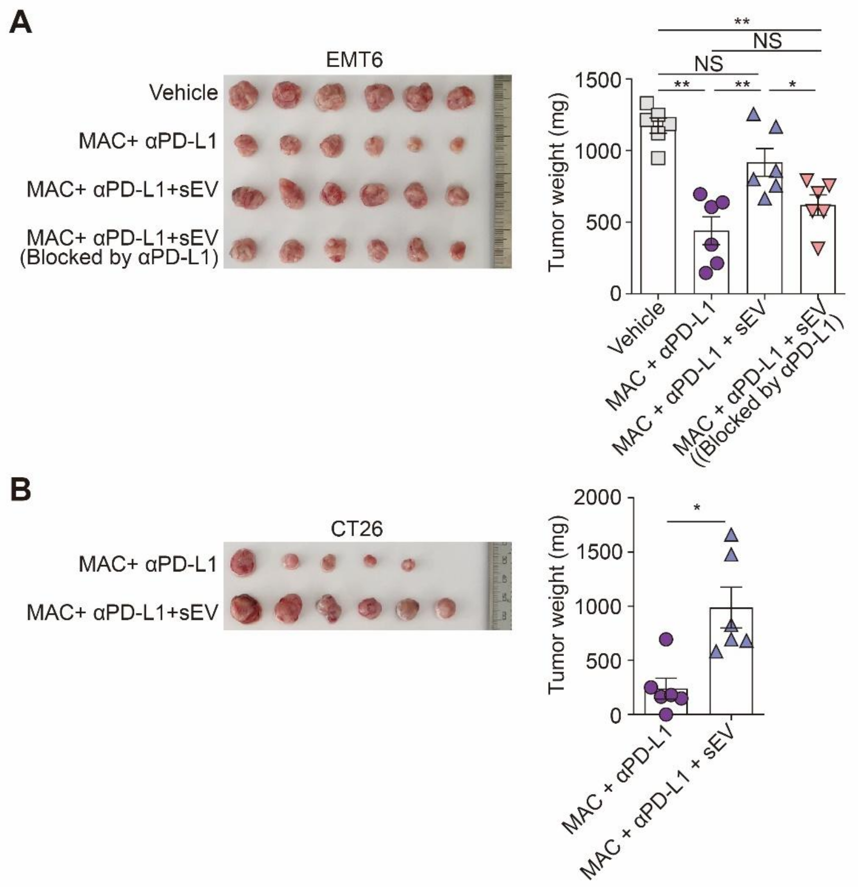

Figure S10. Exogenous EV PD-L1 restore tumor growth suppressed by the combination therapy of MAC and anti-PD-L1 antibody. (A) The tumor images (left) and weights (right) of EMT6 and (B) CT26 tumor models in immunocompetent mice treated with the indicated treatments. The data are presented as means ± SEM ($n = 6$). *$p < 0.05$ and **$p < 0.01$; NS, not significant; Mann–Whitney U test.

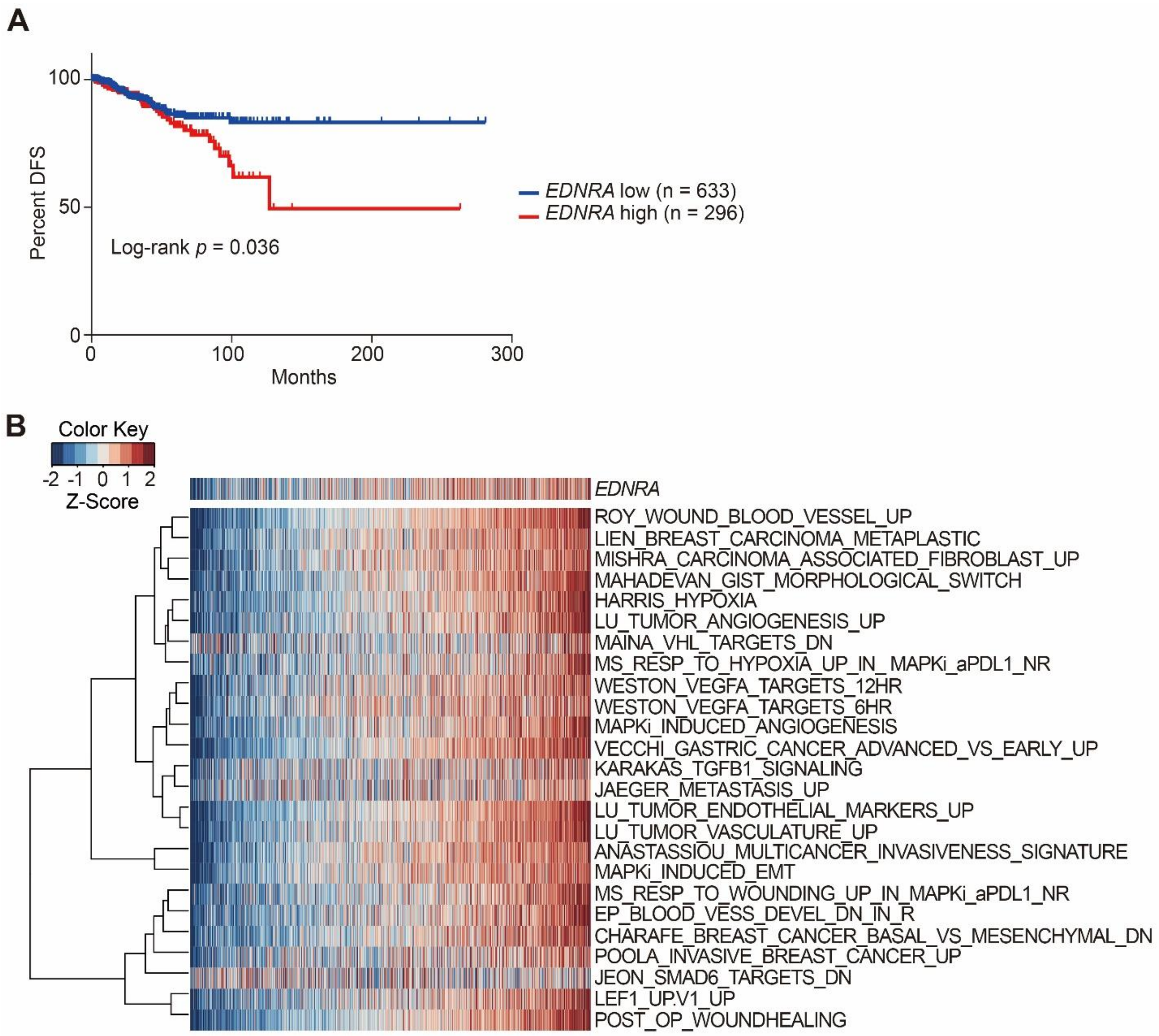

Figure S11. ***EDNRA*** **expression is associated with survival rates and IPRES signature in breast cancer patients.** (A) Disease-free survival (DFS) analysis according to the *EDNRA* expression in the breast cancer patients using the TCGA database (n = 1082, p = 0.036). Each group was divided with an optimized cut-off value (fragments per kilobase transcript per million mapped reads (FPKM) = 8.6). The survival rates were analyzed using the Mantel–Cox test. (B) The heatmap of the IPRES gene signature according to the *EDNRA* expression in 1082 breast cancer patients from the TCGA database.
